# Intracranial Activity of Deruxtecan-Based Antibody–Drug Conjugates in Non-Small Cell Lung Cancer Brain Metastases: A Systematic Review and Meta-Analysis of Prospective Trial Cohorts

**DOI:** 10.3390/cancers18213552

**Published:** 2026-11-05

**Authors:** Tomasz Tykocki, Krzysztof Szalecki

**Affiliations:** 1 Department of Paediatric Neurosurgery, Children’s Hospital Named After Prof. Dr Med. Jan Bogdanowicz, Niekłańska 4/24, 03-924 Warsaw, Poland; 2 Faculty of Medicine, Lazarski University, Świeradowska 43, 02-662 Warsaw, Poland; 3 Department of Neurosurgery, Maria Skłodowska-Curie National Research Institute of Oncology, W.K. Roentgena 5, 02-781 Warsaw, Poland

**Keywords:** antibody–drug conjugate, brain metastases, non-small cell lung cancer, intracranial response, deruxtecan, trastuzumab deruxtecan, patritumab deruxtecan, meta-analysis

## Abstract

Background/Objectives: Brain metastases are common in advanced non-small cell lung cancer (NSCLC). Antibody–drug conjugates (ADCs) have shown intracranial activity in trial subgroups, but intracranial data had rarely been pooled. Methods: MEDLINE, Scopus, Web of Science, registries, and congress sources were searched through 21 September 2026 for prospective trials reporting central nervous system (CNS) outcomes of ADC monotherapy in NSCLC (PROSPERO CRD420261516140). Intracranial objective response rate (icORR) in measurable brain metastases was pooled with a random-intercept logistic model. Risk of bias and GRADE certainty were assessed. Results: Eight prospective cohorts (139 patients; six molecularly selected) of deruxtecan-based ADCs were included. Pooled icORR was 32% (95% CI, 25–41%; 45/139; I^2^ = 0%; prediction interval, 24–43%), and pooled intracranial disease control was 83% (69–92%; five cohorts). In TUXEDO-3, the only dedicated prospective CNS trial, restricted to active metastases, icORR was 30% (6/20). CNS progression-free survival hazard ratios versus chemotherapy were 0.48 (0.23–0.98) and 0.75 (0.53–1.06). Certainty was very low for single-arm response and low for comparative outcomes. Conclusions: Deruxtecan-based ADCs produced intracranial responses in one third of patients with measurable brain metastases from previously treated, predominantly oncogene-driven NSCLC, including active disease. Limited randomized data are compatible with delayed CNS progression. Prospective trials with CNS-specific endpoints are needed.

## Simple Summary

Brain metastases develop in a large proportion of patients with advanced non-small cell lung cancer and are difficult to treat, partly because many systemic drugs reach brain tumours poorly. Antibody–drug conjugates are antibodies linked to a chemotherapy payload, and their large size was long assumed to prevent activity inside the brain. Several recent trials have nevertheless reported shrinkage of brain metastases, but each involved few patients, and the results had not been analyzed together. We systematically searched the literature and combined eight prospective trial cohorts that reported response inside the brain in patients with measurable brain metastases. About one third of patients had their brain metastases shrink, including patients whose metastases were untreated or growing after radiotherapy. In the five cohorts that reported it, about four fifths had their intracranial disease controlled, but many of these patients had stable brain disease when they entered the trials. Most patients had received several previous treatments and had tumours with specific genetic changes, so the results apply mainly to this group. Two randomized trials suggested that brain progression may be delayed compared with chemotherapy. The evidence is limited and uncertain, so these drugs should complement, not replace, surgery and radiotherapy.

## 1. Introduction

Brain metastases occur in up to half of patients with advanced non-small cell lung cancer (NSCLC) and substantially impair survival and quality of life [1,2]. CNS-penetrant tyrosine kinase inhibitors achieve high intracranial response rates in oncogene-driven disease, yet the central nervous system (CNS) remains a frequent site of progression after resistance develops [1]. After failure of targeted therapy or immunotherapy, systemic options with demonstrated intracranial activity are limited.

Surgery, stereotactic radiosurgery, and whole-brain radiotherapy remain the backbone of management, but intracranial relapse after local treatment is frequent, retreatment options are finite, and CNS disease carries a substantial burden in terms of quality of life and health-care resource use [2]. Systemic agents with intracranial activity are therefore attractive as a complement to local therapy, either to treat lesions that are asymptomatic and do not require immediate intervention or to control disease that progresses after irradiation. Historically, however, patients with untreated or symptomatic brain metastases were excluded from registration trials, CNS endpoints were rarely prespecified, and response inside the brain was frequently reported only for exploratory subgroups; consensus guidance has addressed both the eligibility of such patients and the assessment of CNS response [3,4].

Antibody–drug conjugates (ADCs) link a tumour-antigen-directed antibody to a cytotoxic payload. Their molecular size was historically considered incompatible with CNS activity. However, the blood–tumour barrier within metastases is heterogeneously disrupted [5], and newer ADCs carry membrane-permeable topoisomerase I inhibitor payloads released by cleavable linkers, which may permit bystander killing [1,6]. Intracranial responses have been reported with trastuzumab deruxtecan, datopotamab deruxtecan, and patritumab deruxtecan [7,8,9,10,11], while CNS activity has also been explored with other ADCs, including trastuzumab rezetecan [12].

Intracranial activity of an ADC plausibly depends on several sequential steps: delivery of the antibody across a partially disrupted blood–tumour barrier, binding to antigen expressed by metastatic cells, internalization and linker cleavage, and diffusion of the released payload within the tumour microenvironment [5,6]. Deruxtecan-based conjugates share a cleavable tetrapeptide-based linker and a membrane-permeable topoisomerase I inhibitor payload, a combination proposed to underlie killing of neighbouring antigen-negative cells [1,6]. Because these properties are shared by agents directed at different antigens, the intracranial behaviour of this class is of interest beyond any single target, while remaining unproven as a mechanistic explanation in patients.

Most of these observations come from small trial subgroups that were not designed to test CNS efficacy, used heterogeneous criteria, and largely excluded untreated or active metastases [1]. When this review was designed, existing syntheses were narrative [1,13,14], and a recent meta-analysis of ADCs in NSCLC did not synthesize intracranial outcomes [15], whereas intracranial activity of trastuzumab deruxtecan in breast cancer has been quantified by meta-analysis [16]. We therefore pooled trial-level data to estimate intracranial activity of ADCs in NSCLC, to examine whether activity differs by CNS disease status, and to summarize randomized CNS evidence.

The objectives of this review were, first, to estimate the pooled intracranial objective response rate of ADC monotherapy in patients with NSCLC and measurable brain metastases; second, to summarize intracranial disease control, duration of response, and randomized comparisons of CNS outcomes against chemotherapy; third, to examine, descriptively, whether estimates differed by target antigen or by the CNS eligibility criteria of the contributing trials; and fourth, to appraise risk of bias and certainty of evidence so that the size of the effect and the confidence that can be placed in it are reported separately.

## 2. Materials and Methods

### 2.1. Protocol, Registration, and Reporting

This systematic review and meta-analysis of published trial-level data was conducted and reported in accordance with PRISMA 2020 [17]; the completed PRISMA 2020 checklist is provided in File S1. The review protocol was registered retrospectively in PROSPERO (CRD420261516140). Study selection, data extraction, and risk-of-bias assessment were performed by one reviewer (T.T.), reflecting the resources available to a two-author review; the final full-text eligibility decisions were independently re-screened by a second reviewer (K.S.).

To limit single-reviewer error, every numeric value entering an analysis was verified manually against the source document (Section 2.4); duplicate independent data extraction and appraisal were not performed, and this is acknowledged as a limitation.

### 2.2. Eligibility Criteria

The target population was adults with advanced NSCLC and brain metastases not requiring immediate local therapy. Eligible sources were prospective phase I–III trials of ADC monotherapy in adults with advanced NSCLC reporting any CNS-specific efficacy outcome. The primary quantitative synthesis was restricted to cohorts reporting intracranial response counts in patients with measurable brain metastases. Randomized comparisons of CNS outcomes, responses in non-measurable disease, and time-to-event CNS outcomes were synthesized separately. Systemic response in patients with brain metastases was not used as a surrogate for intracranial response. Combination regimens and concurrent radiotherapy were excluded. Retrospective cohorts were not eligible for the primary analysis but were used in a post hoc observational sensitivity analysis if they reported intracranial response separately for patients who received no local CNS therapy during or immediately before ADC treatment, as defined by the study authors.

### 2.3. Information Sources and Search

MEDLINE via PubMed, Scopus, and Web of Science Core Collection were searched from database inception through 21 September 2026, without language restrictions. The search strategies used combinations of terms for non-small cell lung cancer, antibody–drug conjugates and individual ADCs, and CNS/brain metastases; full electronic search strategies for all databases are provided in File S2. ClinicalTrials.gov, Europe PMC/PubMed Central, reference lists of relevant reviews [1,13,14,15], and ASCO Annual Meeting, ESMO Congress, and IASLC World Conference on Lung Cancer abstracts and presentations (generic concept and trial-name searches, without date restriction, through 21 September 2026) were hand-searched as supplementary sources, in part to compensate for the absence of Embase (File S2).

### 2.4. Study Selection and Data Extraction

After deduplication, records underwent title/abstract screening followed by full-text eligibility assessment according to the criteria above, both performed by one reviewer (T.T.). In a second screening round, the second reviewer (K.S.) independently reviewed the final full-text eligibility decisions, including the reasons for exclusion; any disagreements were resolved by discussion. The seven contributing reports are listed in Table 1 [11,18,19,20,21,22,23]; HERTHENA-Lung02 response categories were extracted from the presentation slides [24]. For each cohort we extracted design, ADC, target, dose, population, CNS status (untreated, treated/stable, or progressing after local therapy), prior local treatment, imaging modality, response criteria, assessor, the number of patients with measurable brain metastases and intracranial responders, disease control, and CNS time-to-event outcomes. Safety outcomes (grade ≥ 3 adverse events, adjudicated interstitial lung disease [ILD]/pneumonitis, and treatment-related deaths) and corticosteroid eligibility criteria or use were extracted from CNS-subgroup reports where available and otherwise from the parent-trial publication, and labelled accordingly. Cohorts were mapped to trials so that no patient contributed more than once. Counts inferred from lesion-classification rules were labelled reconstructed (the derivation of each is shown in File S6); counts calculated from reported percentages or response categories were labelled derived; all other values were directly reported. Data were extracted by one reviewer (T.T.) with assistance from large language models, which were used to locate and tabulate candidate values in source documents but not to make eligibility, risk-of-bias, or certainty judgements. Every numeric field entering an analysis (denominators, responder and disease-control counts, best-overall-response categories, hazard ratios and confidence intervals, and safety counts) was checked manually against the published abstract, full text, or presentation slide, and study-level confidence intervals were recalculated and compared with published values.

Congress abstracts and presentation slides were treated as reports of the corresponding trial rather than as separate studies. Where several reports described the same trial, one estimate per outcome was used according to a hierarchy: a dedicated peer-reviewed CNS analysis, then the primary peer-reviewed publication, then the most recent congress report. Cohorts defined by dose within a single report were treated as independent because they comprised different patients.

### 2.5. Outcomes and Definitions

The primary outcome was intracranial objective response rate (icORR), as defined in each source, among patients with measurable brain metastases. Secondary outcomes were intracranial disease control rate (icDCR), the risk ratio for intracranial response and the hazard ratio (HR) for CNS progression-free survival (CNS-PFS) in randomized comparisons, and descriptive CNS duration of response. CNS-PFS definitions were recorded; in TROPION-Lung01 the endpoint included death from any cause [20], whereas the HERTHENA-Lung02 definition was not reported in the available presentation [24]. Response criteria (CNS RECIST or Response Assessment in Neuro-Oncology Brain Metastases [RANO-BM] [3]) and assessors were recorded.

Measurable brain metastases were defined by the criteria applied in each trial, which generally require at least one target lesion measuring 10 mm or more [3]. Denominators were those reported by each source and included patients without evaluable post-baseline CNS imaging, who were counted as non-responders; this conservative approach avoids selecting an evaluable population defined after treatment had started. Intracranial disease control was defined as complete response, partial response, or stable disease within the brain. Responses in patients with non-measurable CNS disease were tabulated separately and were never combined with measurable-disease estimates, because response in non-target lesions is less reliably assessed.

### 2.6. Risk of Bias and Certainty of Evidence

Single-arm intracranial estimates were appraised with the eight non-comparative items of the Methodological Index for Non-Randomized Studies (MINORS; maximum 16) [25]. Items that could not be verified from available sources were recorded as not verified rather than scored as inadequate. MINORS was used as a structured appraisal framework for non-comparative CNS cohorts, including single-arm CNS subsets derived from randomized trials, rather than as a tool specifically validated for post hoc trial subgroups.

Randomized CNS comparisons were appraised with the revised Cochrane risk-of-bias tool (RoB 2) [26], separately for intracranial response and CNS-PFS, using signalling questions. Certainty was rated per outcome using GRADE [27] against the target population defined above; non-comparative proportions started at low certainty and randomized comparisons at high certainty. All assessments were performed by one reviewer (T.T.); detailed judgements are provided in File S3.

### 2.7. Statistical Analysis

Proportions were pooled with a random-intercept logistic regression model (binomial–normal generalized linear mixed model, GLMM) fitted by maximum likelihood with 40-point Gauss–Hermite quadrature [28], which uses exact binomial likelihoods and avoids arcsine back-transformation problems [29]. Study-level 95% confidence intervals used the Clopper–Pearson method [30]. Heterogeneity statistics (Cochran’s Q, I^2^ [31], τ^2^ by restricted maximum likelihood) and the 95% prediction interval (t distribution, k − 2 degrees of freedom) were derived from an inverse-variance random-effects analysis of logit proportions, whose weights are displayed in forest plots because the GLMM does not assign weights.

All subgroup and sensitivity analyses were post hoc and exploratory. Subgroups were target antigen and eligibility restriction (cohorts limited to untreated or progressing metastases versus cohorts with mixed eligibility); eligibility restriction describes trial design and does not separate active from stable disease. Sensitivity analyses comprised inverse-variance random-effects models of logit proportions with REML estimation of τ^2^ and the Hartung–Knapp–Sidik–Jonkman adjustment [32], unmodified and modified (variance factor truncated at 1) [33]; leave-one-out analysis; exclusion of reconstructed response counts (HERTHENA-Lung01, HERTHENA-Lung02); restriction to peer-reviewed reports; restriction to independent central review (omitting TUXEDO-3); and addition of observational evidence from the TRACER/HERTras real-world cohort [34], first restricted to patients without local CNS therapy during or immediately before trastuzumab deruxtecan and then, as a potentially confounded analysis, including all patients with measurable metastases (File S4).

For randomized comparisons, study-level risk ratios were presented without pooling because only two trials, with different comparators and one zero-event control arm, were available. Trial-level CNS-PFS HRs are presented as the comparative result. An exploratory common-effect inverse-variance pooled HR (DerSimonian–Laird random-effects as sensitivity) is reported only in File S4 (Figure S2), because the trials differ in ADC, comparator, and population, so a common effect cannot be assumed.

Small-study effects for the primary outcome were explored with a funnel plot of logit-transformed icORR against its standard error, with pseudo-95% confidence limits around the pooled estimate, and with Egger regression (intercept tested with a t distribution, k − 2 degrees of freedom). Because the logit of a proportion and its standard error are mathematically correlated, a complementary regression of the logit proportion on the inverse of sample size (weighted by inverse variance) was also performed. These analyses were post hoc and exploratory, because fewer than 10 cohorts were available, below the minimum generally recommended for asymmetry tests [35] (File S5). Analyses used Python (version: 3.12) with SciPy 1.17.1 and NumPy 2.4.4 [36].

Further sensitivity analyses were added during peer review. Because an estimate of τ^2^ = 0 from eight small cohorts does not establish homogeneity, the GLMM was refitted with τ^2^ fixed at the upper limit of its Q-profile 95% confidence interval [37], with the prediction interval recalculated, and a Bayesian binomial–normal model with half-normal priors on τ (scales 0.5 and 1.0) was fitted [38]. Because the HERTHENA-Lung01 and HERTHENA-Lung02 measurable-disease numerators were derived from response categories, pooled icORR and the HERTHENA-Lung02 risk ratio were recalculated under scenarios with lower or higher numerators (File S6). The icDCR analysis was repeated after excluding the cohort with 100% disease control (DESTINY-Lung05).

Deviations from the registered protocol are reported here in full. The protocol was registered in PROSPERO after data extraction had started, and the review is therefore retrospectively registered. All subgroup and sensitivity analyses were post hoc, including the analyses by target antigen and by CNS eligibility restriction, and the observational sensitivity analysis incorporating real-world data was added during manuscript revision. The analyses described in the preceding paragraph and the safety extraction were added during peer review and are not part of the registered protocol. No outcome was excluded from reporting because of its direction or statistical significance.

## 3. Results

### 3.1. Study Selection

**Figure 1:**
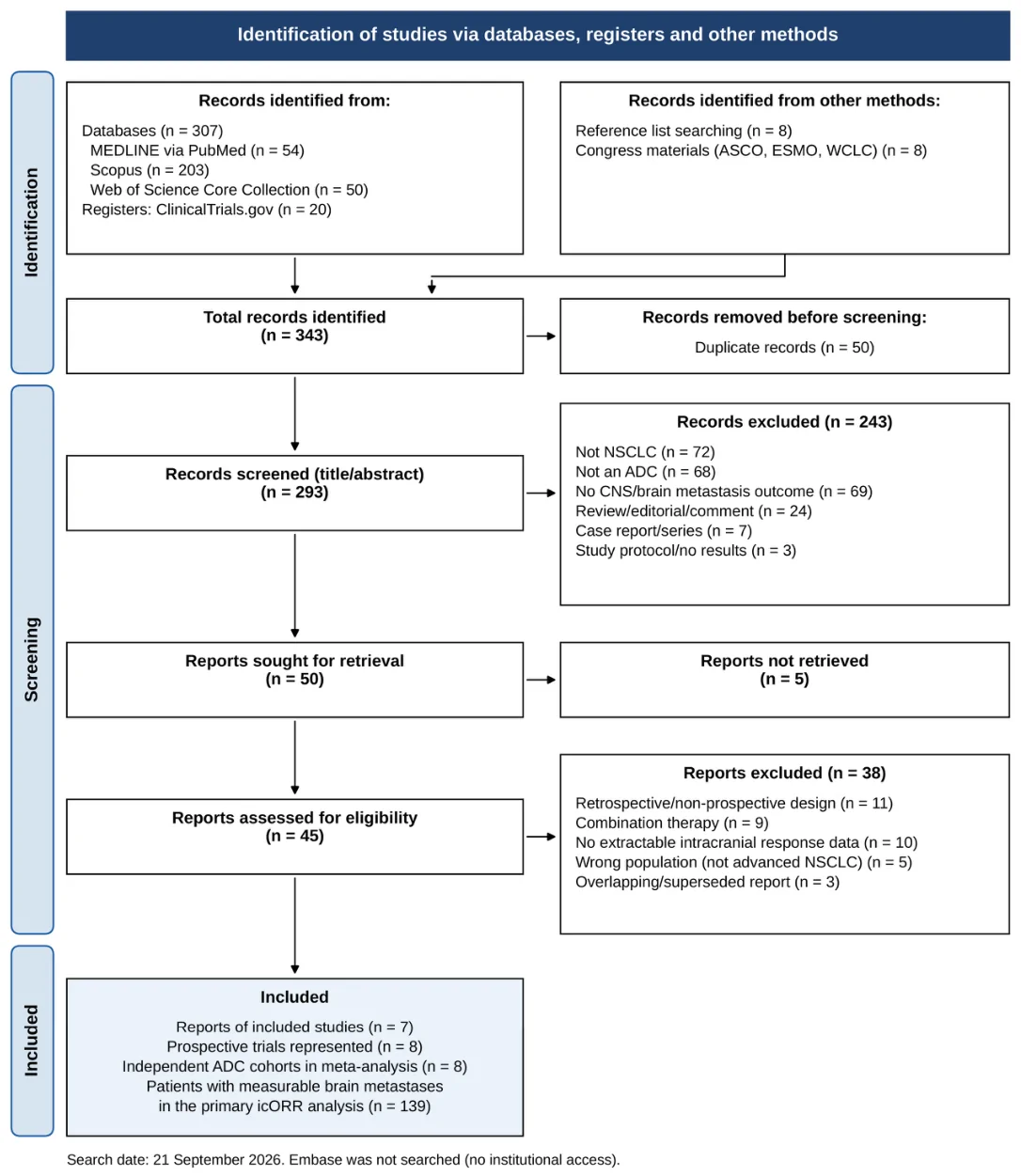
PRISMA 2020 flow diagram of study identification and selection. Records from all sources were deduplicated together before screening.

The search identified 343 records (307 from databases, 20 from ClinicalTrials.gov, and 16 from other methods), of which 50 duplicates were removed, leaving 293 records for screening. After exclusion of 243 records, 50 reports were sought for full-text review; five were not retrieved and 45 were assessed for eligibility. Following exclusion of 38 full-text reports, seven reports representing eight prospective trials and eight independent ADC cohorts were included, comprising 139 patients with measurable brain metastases (Figure 1).

### 3.2. Characteristics of Included Cohorts

The Jänne report defined cohorts by dose, pooling DESTINY-Lung01 and DESTINY-Lung02 for 6.4 mg/kg; the included cohorts were therefore trastuzumab deruxtecan 5.4 mg/kg (n = 14) and 6.4 mg/kg (n = 30) from DESTINY-Lung01/02 [18] and 5.4 mg/kg from DESTINY-Lung05 in Chinese patients with pretreated, asymptomatic or neurologically stable metastases (n = 10) [23]; datopotamab deruxtecan from TROPION-Lung05 (n = 18) [19] and TROPION-Lung01 (n = 16) [20]; and patritumab deruxtecan from HERTHENA-Lung01 (n = 7) [11], HERTHENA-Lung02 (n = 24) [21], and TUXEDO-3 (n = 20) [22] (Table 1). All ADCs carried a deruxtecan payload. TUXEDO-3 was the only dedicated prospective CNS trial and enrolled active metastases (seven untreated, 13 progressing after local therapy) with at least one brain lesion ≥10 mm on MRI and no need for immediate local treatment, assessed locally by RANO-BM [22]. TROPION-Lung01 also provided a post hoc analysis restricted to untreated or progressing brain metastases, whereas the remaining trial cohorts predominantly required stable or asymptomatic CNS disease and were assessed by independent central review.

The measurable-disease estimates for HERTHENA-Lung01 (2/7) and HERTHENA-Lung02 (7/24) were reconstructed because all or most intracranial complete responses occurred in patients with non-target lesions only [11,24]. Trastuzumab rezetecan was not included because HORIZON-Lung reported systemic rather than intracranial response in patients with brain metastases [12]. The TRACER/HERTras real-world cohort (retrospective, 68 centres) reported intracranial response in 27 patients with measurable metastases, 18 of whom received no local CNS therapy during or immediately before trastuzumab deruxtecan [34]. MINORS appraisals are shown in File S3 (Table S1A).

Across the eight cohorts, three were HER2-directed (trastuzumab deruxtecan; 54 patients), two TROP2-directed (datopotamab deruxtecan; 34 patients), and three HER3-directed (patritumab deruxtecan; 51 patients). Six cohorts enrolled molecularly selected populations (HER2-mutant, EGFR-mutant, or other actionable genomic alterations) and two were molecularly unselected. Two cohorts derived from randomized phase 3 trials, five from single-arm or dose-defined phase 2 cohorts, and one from a dedicated CNS trial. Four cohorts were reported only in congress materials, and CNS response was assessed by independent central review in all cohorts except TUXEDO-3, in which local investigators applied RANO-BM criteria. Prior brain radiotherapy, imaging modality, and follow-up, where reported, are shown in Table 1; in the trastuzumab deruxtecan cohorts, 9 of 14 and 10 of 30 patients were assessed by computed tomography rather than magnetic resonance imaging [18].

### 3.3. Intracranial Objective Response

**Figure 2:**
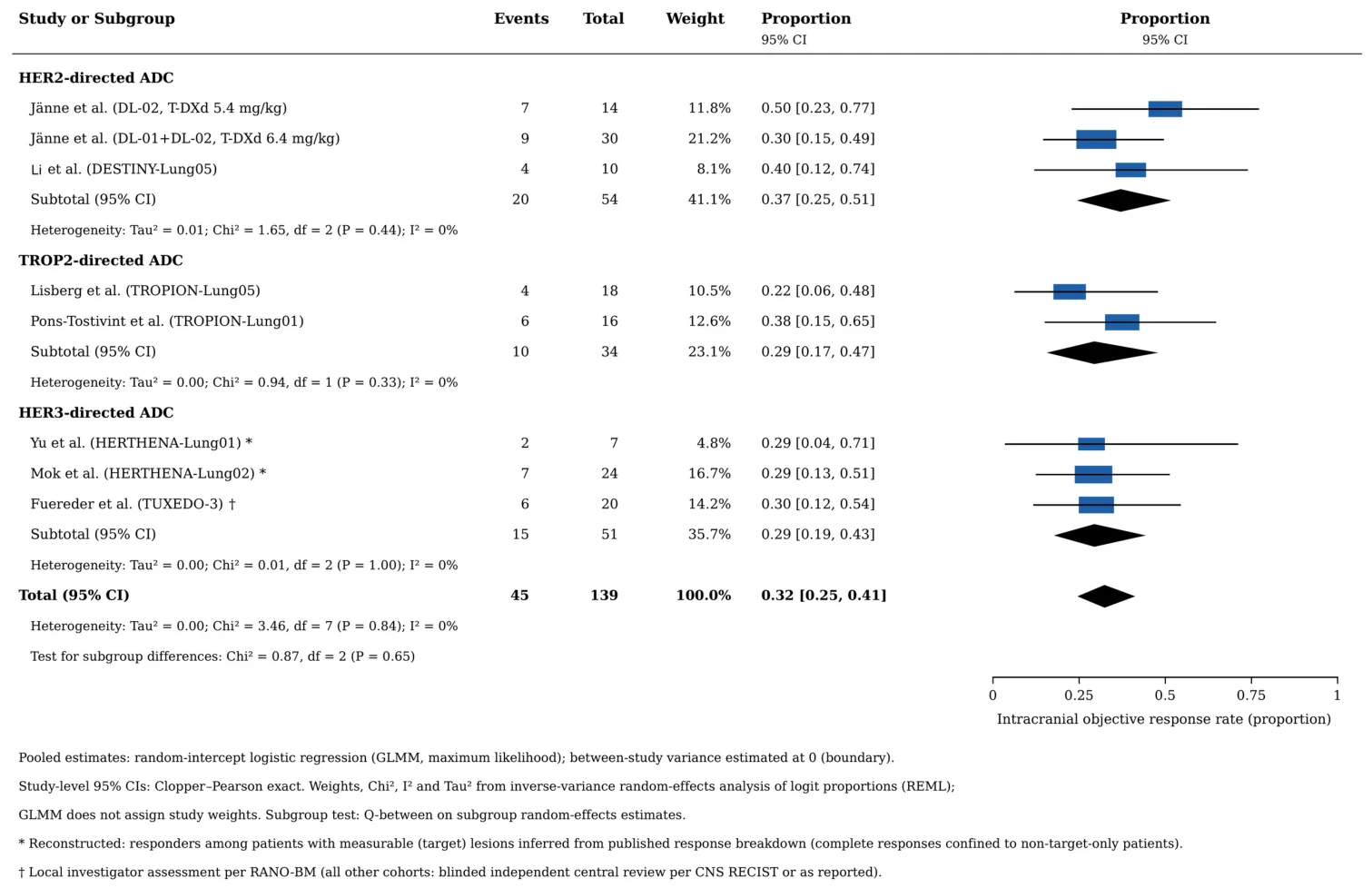
Forest plot of intracranial objective response rate with deruxtecan-based antibody–drug conjugates in patients with NSCLC and measurable brain metastases, by target antigen. The pooled estimate was obtained using a random-intercept logistic regression model (GLMM); study-level 95% confidence intervals were calculated using the Clopper–Pearson method. Displayed weights and heterogeneity statistics are from an inverse-variance random-effects analysis of logit proportions. Data are from the included cohorts [11,18,19,20,21,22,23].

Cohort icORRs ranged from 22% to 50% (Table 2). The pooled icORR was 32% (95% CI, 25–41%; 45/139), with τ^2^ estimated at zero (Q = 3.46, df = 7, *p* = 0.84; I^2^ = 0%) and a 95% prediction interval of 24–43% (Figure 2). The Q-profile 95% confidence interval for τ^2^ was 0 to 0.31; with τ^2^ fixed at 0.31, pooled icORR was 32% (95% CI, 22–45%) with a prediction interval of 9–69%, and Bayesian models with half-normal priors gave 32% (24–42%) under both prior scales (File S4, Table S2). The point estimate was therefore stable, but the range of effects to be expected in a new cohort is considerably wider than the zero-heterogeneity model suggests. Exploratory estimates by target antigen were 37% (95% CI, 25–51%) for HER2-, 29% (17–47%) for TROP2-, and 29% (19–43%) for HER3-directed ADCs (*p* = 0.65); target antigen is confounded by molecular subtype and treatment history. In TUXEDO-3, the only dedicated prospective CNS trial restricted to active brain metastases, icORR was 30% (6/20), with responses in 2 of 7 untreated and 4 of 13 progressing patients; median intracranial PFS was 4.8 months [22]. Cohorts with eligibility restricted to untreated or progressing metastases and cohorts with mixed eligibility gave similar descriptive estimates (33% and 32%; File S4, Table S2).

### 3.4. Sensitivity Analyses

Hartung–Knapp models gave 33% (95% CI, 26–40%; unmodified) and 33% (24–43%; modified). Across prospective-only analyses, pooled icORR ranged from 30% to 34%, and no single cohort changed the estimate by more than 2 percentage points (File S4, Table S2 and Figure S1). Excluding reconstructed counts gave 33% (95% CI, 25–43%), and restricting to peer-reviewed reports gave 34% (24–46%). Adding TRACER/HERTras patients without local CNS therapy (11/18) increased the estimate to 36% (28–44%; 56/157) with little heterogeneity (I^2^ = 7%). Adding the whole TRACER/HERTras measurable cohort (20/27) increased it to 38% (27–50%) and introduced substantial heterogeneity (I^2^ = 54%; *p* = 0.03), consistent with confounding by concomitant local therapy, since all nine locally treated patients responded. Because CNS RECIST does not allow a partial response in patients with non-target lesions only, the HERTHENA-Lung01 count (one complete and one partial response among seven patients with target lesions) and the HERTHENA-Lung02 counts (all seven partial responses in each arm, with every complete response in a non-target-only patient) follow from the reported response categories (File S6). Under deliberately extreme alternatives (HERTHENA-Lung01 0/7 to 4/7; HERTHENA-Lung02 4/24 to 12/24), pooled icORR ranged from 29% (95% CI, 22–37%) to 37% (30–46%); varying each HERTHENA-Lung02 arm by two responders gave risk ratios of 0.65 to 2.10, all with confidence intervals including 1.

### 3.5. Disease Control, Durability, and Randomized Evidence

**Figure 3:**
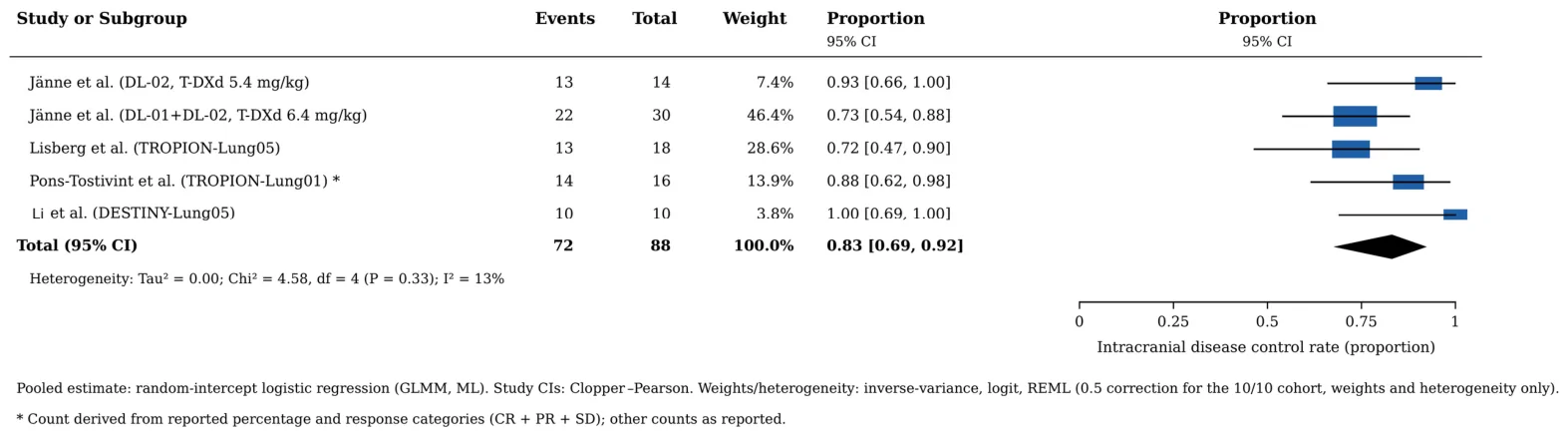
Forest plot of intracranial disease control rate with deruxtecan-based antibody–drug conjugates in patients with NSCLC and measurable brain metastases. The pooled estimate was obtained using a random-intercept logistic regression model, with study-level Clopper–Pearson 95% confidence intervals. Data are from the included cohorts [18,19,20,23].

The pooled icDCR in five cohorts was 83% (95% CI, 69–92%; 72/88; I^2^ = 13%) (Figure 3); the inverse-variance estimate was 79% (62–90%), the difference reflecting the 10/10 DESTINY-Lung05 cohort, and excluding that cohort gave 79% (69–87%). Of the 72 patients with disease control, 30 had an objective response and 42 had stable disease as best intracranial response; because most of these cohorts enrolled stable or asymptomatic metastases, stable disease cannot be attributed to treatment with confidence. Median intracranial duration of response was 9.5 and 4.4 months with trastuzumab deruxtecan 5.4 and 6.4 mg/kg [18], 8.4 months in HERTHENA-Lung01 [11], and 4.5 versus 4.2 months with patritumab deruxtecan versus chemotherapy in HERTHENA-Lung02 [21].

**Figure 4:**
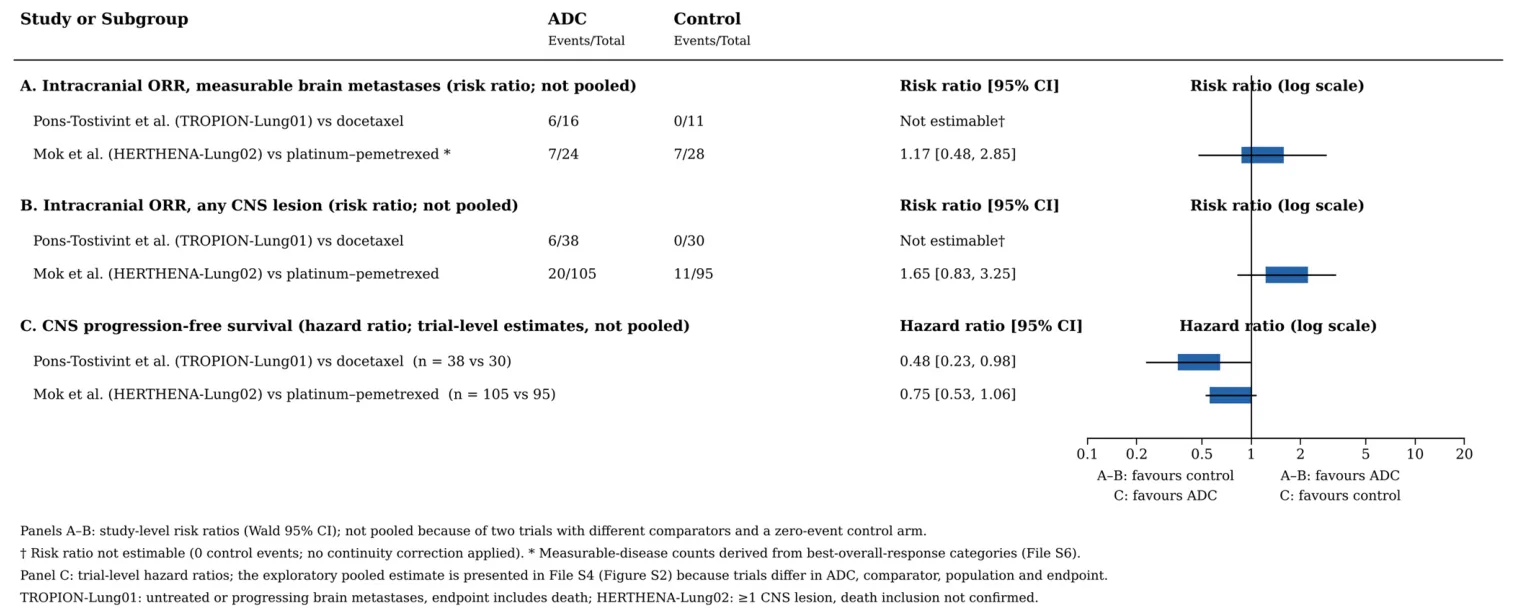
Forest plot of randomized CNS outcomes with deruxtecan-based antibody–drug conjugates versus chemotherapy. Panels A and B show study-level risk ratios for intracranial objective response in patients with measurable brain metastases and in patients with any CNS lesion, respectively; response estimates were not pooled because only two trials with different comparators were available and one control arm contained zero events. Panel C shows trial-level hazard ratios for CNS progression-free survival, which were not pooled in the main analysis because the trials differed in ADC, comparator, CNS population, and endpoint definition (exploratory pooled estimate in File S4, Figure S2). Data are from the included randomized trials [20,21,24].

Two randomized trials reported CNS outcomes (Figure 4). In TROPION-Lung01, 6 of 16 patients with measurable untreated or progressing metastases responded with datopotamab deruxtecan versus 0 of 11 with docetaxel [20]. In HERTHENA-Lung02, reconstructed measurable-disease response was 7 of 24 versus 7 of 28 with patritumab deruxtecan versus platinum–pemetrexed (risk ratio, 1.17; 95% CI, 0.48–2.85), and 20 of 105 versus 11 of 95 among all patients with CNS lesions (risk ratio, 1.65; 95% CI, 0.83–3.25) [24]. CNS-PFS HRs were 0.48 (95% CI, 0.23–0.98) in TROPION-Lung01 (untreated or progressing metastases, n = 68) and 0.75 (0.53–1.06) in HERTHENA-Lung02 (any CNS lesion, n = 200). An exploratory pooled estimate is given in File S4 (Figure S2). RoB 2 judgements, made separately by outcome, are shown in File S3 (Table S1B).

### 3.6. Risk of Bias, Small-Study Effects, and Certainty of Evidence

**Figure 5:**
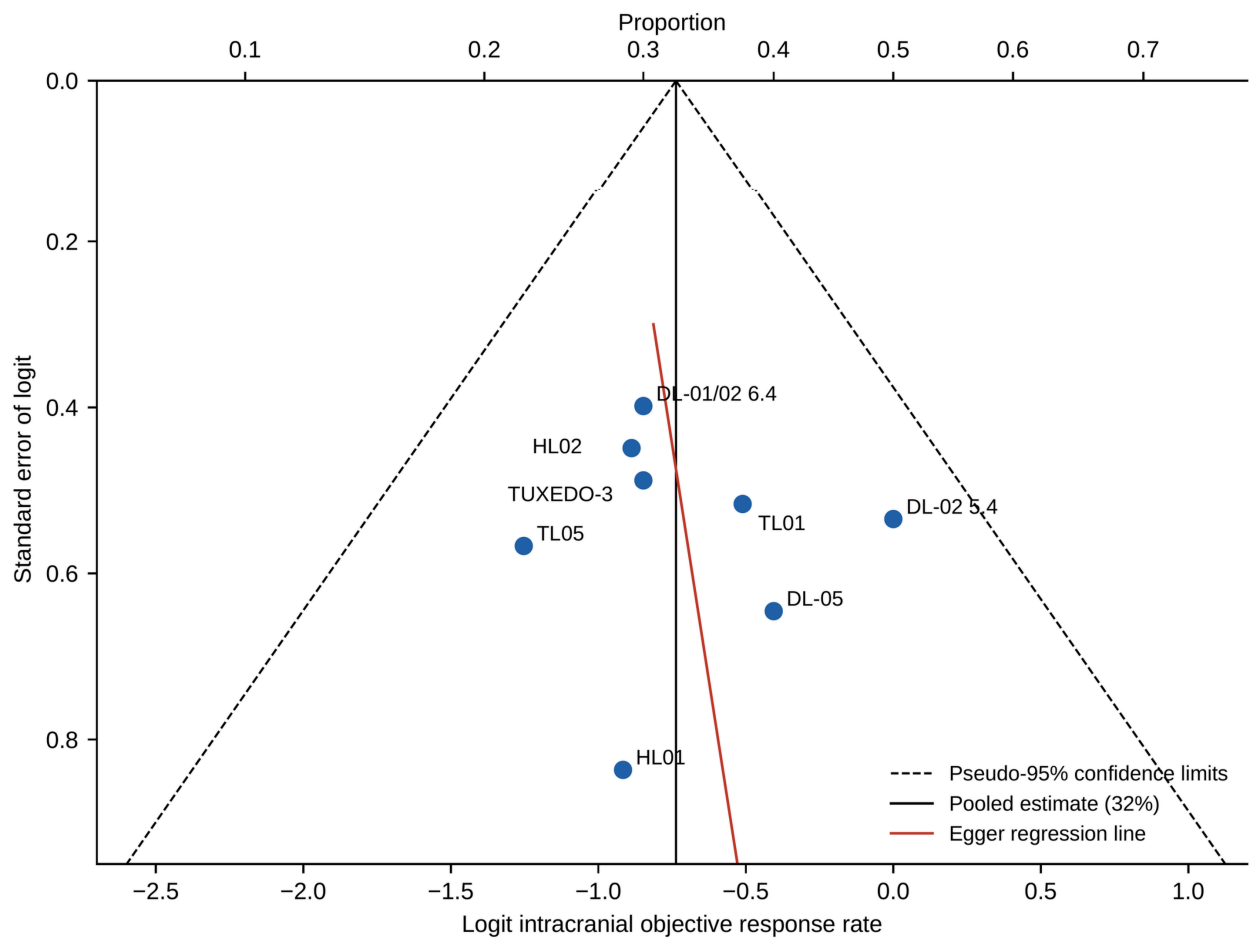
Funnel plot of logit-transformed intracranial objective response rate against its standard error for eight prospective ADC cohorts. Dashed lines, pseudo-95% confidence limits around the pooled estimate; solid vertical line, pooled estimate; red line, Egger regression line (intercept 0.44, 95% CI −2.88 to 3.76; t = 0.32, df = 6, *p* = 0.76). Fewer than the 10 cohorts generally recommended for asymmetry testing were available. DL, DESTINY-Lung; HL, HERTHENA-Lung; TL, TROPION-Lung.

Most single-arm CNS analyses were post hoc subsets, two counts were reconstructed, four cohorts were available only as congress abstracts or presentations, and non-evaluable patients were counted as non-responders. Among randomized comparisons, intracranial response in TROPION-Lung01 was at high risk of bias because of imbalanced missing CNS assessments, and the remaining comparisons raised some concerns. Funnel-plot analyses showed no marked asymmetry (Egger intercept 0.44, 95% CI −2.88 to 3.76; *p* = 0.76; sample-size-based regression *p* = 0.43) (Figure 5), but were underpowered with eight cohorts and cannot exclude selective reporting of favourable CNS subgroups, which operates within trials rather than across them. Certainty was very low for single-arm icORR and icDCR, and low for comparative response and CNS-PFS (Table 3; File S3). Because GRADE was developed for comparative effects, ratings for single-arm proportions should be read together with the risk-of-bias appraisal.

In the MINORS appraisal, items relating to the statement of aim, prospective data collection, appropriateness of the endpoint, and unbiased endpoint assessment were generally adequate, whereas prospective sample-size calculation for the CNS analysis was absent in every cohort except TUXEDO-3, and follow-up duration or loss to follow-up could often not be verified from the available reports (File S3, Table S1A). Downgrading of certainty was driven by risk of bias for the single-arm outcomes and by risk of bias together with imprecision for the comparative outcomes; inconsistency and indirectness were not serious relative to the target population defined a priori, and publication bias was not used as a reason to downgrade because it could not be assessed reliably.

### 3.7. Safety

CNS-subgroup safety data were available for the trastuzumab deruxtecan cohorts and TUXEDO-3; for the other cohorts only parent-trial data were available (Table 4). In DESTINY-Lung01/02, grade ≥ 3 adverse events occurred in 65% and 76% of patients with brain metastases at 5.4 and 6.4 mg/kg, and adjudicated drug-related ILD in 16% and 20%, with one drug-related death in each dose group [18]. In TUXEDO-3, treatment-related adverse events occurred in 80% of patients, with grade 3–4 neutropenia in 15% and febrile neutropenia in 10% and no treatment-related deaths [22]. In the parent trials, adjudicated drug-related ILD occurred in 3.6% (TROPION-Lung05), 5.3% and 5.2% (HERTHENA-Lung01 and -Lung02), 8.8% (TROPION-Lung01), and 12.9–28.0% (trastuzumab deruxtecan) of patients, with grade 5 ILD in 0.4–0.7% of patients receiving datopotamab or patritumab deruxtecan [7,8,9,10,11,24]. DESTINY-Lung01/02, HERTHENA-Lung01, and HERTHENA-Lung02 excluded patients requiring corticosteroids for brain metastases [11,18,24], and HERTHENA-Lung01 reported that corticosteroid and neurological-symptom data needed for RANO-BM were not routinely collected [11]; on-treatment corticosteroid use was not reported for any CNS subgroup.

## 4. Discussion

### 4.1. Principal Findings

In eight prospective cohorts, deruxtecan-based ADCs produced intracranial responses in about one-third of patients with measurable NSCLC brain metastases and disease control in about four-fifths, and two randomized trials provided limited evidence compatible with delayed CNS progression relative to chemotherapy. At our search cutoff, the database and supplementary searches identified narrative syntheses [1,13,14] and an NSCLC ADC meta-analysis addressing systemic outcomes [15], but no quantitative synthesis of intracranial ADC outcomes in NSCLC. One week after that cutoff, Zhou et al. published a meta-analysis of systemic and intracranial ADC activity in NSCLC with brain metastases (13 studies; 905 patients with brain metastases) that reported an intracranial ORR of 33% (95% CI, 25–43%) and an intracranial disease control rate of 78% [39]. The present review is therefore not the first quantitative synthesis of this question. Its estimates agree closely with those of Zhou et al., and it adds a restriction to prospective trial cohorts, a uniform measurable-disease denominator with explicit labelling of derived counts, separation of single-arm from randomized evidence, and an explicit certainty rating that is very low for single-arm response.

### 4.2. Activity in Active Brain Metastases

A key clinical question for neuro-oncology is whether activity extends to active disease. TUXEDO-3 provides the most direct prospective evidence in active CNS disease because it was specifically designed for this population. The trial enrolled untreated or progressing metastases and observed a 30% intracranial response rate, including responses in lesions progressing after local therapy [22]. TROPION-Lung01 also demonstrated intracranial responses in a post hoc subset restricted to untreated or progressing brain metastases, although interpretation is limited by the small sample size and missing CNS imaging [20]. Real-world data point in the same direction: in TRACER/HERTras, 11 of 18 patients with measurable metastases who received no local CNS therapy responded intracranially, although retrospective response assessment without central review may overestimate activity [34]. The remaining trial cohorts predominantly enrolled patients with stable or asymptomatic CNS disease. The included evidence does not encompass patients requiring immediate local therapy; therefore, these findings do not support deferring radiosurgery or surgery for symptomatic or rapidly progressing lesions.

### 4.3. Randomized CNS Evidence

Randomized evidence refines rather than confirms the single-arm estimates. In HERTHENA-Lung02, the only phase 3 trial with a prespecified CNS endpoint in the post-third-generation EGFR inhibitor setting, intracranial response in measurable disease was similar between arms, whereas response in non-target lesions and intracranial PFS favoured patritumab deruxtecan [21]. Platinum–pemetrexed has intracranial activity of its own, and the modest CNS-PFS advantage, together with the absence of overall survival benefit in that trial [21], tempers expectations. The larger contrast in TROPION-Lung01 against docetaxel derives from a small post hoc subset with substantial missing CNS imaging [20]. The two trials differ in ADC, comparator, and population, and the TROPION-Lung01 endpoint included death; the pooled HR is therefore an exploratory summary of low certainty, not evidence of CNS-specific superiority for any agent.

### 4.4. Durability, Imaging, and Endpoint Definitions

Durability and imaging quality limit interpretation of the response data. Where reported, median intracranial duration of response ranged from 4.4 to 9.5 months, and several HERTHENA-Lung01 responses were determined on computed tomography alone, while complete responses in non-target lesions dominated the all-lesion estimates [11,21]. For this reason, measurable disease was used for the primary analysis, consistent with guidance for CNS trial design [3,4]. Neurological function, symptom burden, and neurocognitive outcomes were rarely reported in extractable form and should become standard CNS endpoints.

### 4.5. Mechanistic Considerations and Safety

Similar estimates across HER2-, TROP2-, and HER3-directed agents are compatible with a payload-driven mechanism operating across a disrupted blood–tumour barrier [1,5,6], but target antigen is entangled with molecular subtype, treatment history, and eligibility, and payload could not be separated from target. The target comparison is exploratory and does not imply comparable efficacy of individual agents. CNS-subgroup safety data were limited to the trastuzumab deruxtecan cohorts and TUXEDO-3 (Table 4); in DESTINY-Lung01/02, grade ≥ 3 adverse events were numerically more frequent in patients with than without brain metastases [18]. ILD, which occurred in 16–20% of patients with brain metastases receiving trastuzumab deruxtecan [18] and in 3.6–8.8% of patients in the parent trials of the other conjugates [9,10,11,24], and hematological toxicity should be weighed when sequencing ADCs with local therapy, and the safety of concurrent radiosurgery was outside the scope of this analysis.

### 4.6. Clinical Implications

For neurosurgical and neuro-oncological practice, these data support considering a deruxtecan-based ADC as part of multidisciplinary planning in patients whose brain metastases are asymptomatic or stable, or are progressing after irradiation without requiring immediate decompression or re-irradiation, particularly when the systemic indication for the ADC is already established. They do not support withholding surgery or radiosurgery from patients with symptomatic, haemorrhagic, or rapidly progressing lesions, or from those requiring corticosteroids for mass effect, because such patients were not represented in the included trials. Disease control should not be equated with tumour regression: stable disease accounted for more than half of the pooled disease control, in cohorts that largely enrolled stable or asymptomatic metastases, so only the response rate provides direct evidence of intracranial antitumour activity. A pooled response rate of approximately one third also implies that most patients will not respond intracranially, so close radiological surveillance with magnetic resonance imaging and predefined criteria for salvage local therapy remain necessary. Sequencing considerations include the pulmonary and hematological toxicity profile of deruxtecan conjugates, the absence of data on concurrent radiosurgery, and the practical need to distinguish true intracranial progression from post-treatment imaging changes when local therapy has preceded ADC treatment.

### 4.7. Implications for Research

The main methodological lesson of this synthesis is that CNS efficacy is being estimated from data that were not designed to provide it. Future trials enrolling patients with brain metastases should prespecify intracranial endpoints with measurable-disease denominators, apply RANO-BM or CNS RECIST criteria with central review, mandate baseline and on-treatment magnetic resonance imaging at fixed intervals, and report outcomes separately for untreated, stable, and post-irradiation progressive disease, together with corticosteroid use and neurological function. Dedicated CNS cohorts, as in TUXEDO-3, remain the most informative design and should be embedded in the development of new conjugates rather than added retrospectively. Questions that this review cannot answer, and that merit prospective study, include the activity of ADCs in leptomeningeal disease, the safety and efficacy of concurrent or sequential radiosurgery, the value of antigen expression in metastatic rather than primary tissue as a predictive biomarker, and whether intracranial response translates into neurological benefit. An individual-participant-data meta-analysis, if sponsors made CNS subsets available, would resolve several of the denominator and definition problems encountered here.

### 4.8. Strengths and Limitations

Strengths of this review include a registered (albeit retrospectively) protocol, a reproducible multi-database search, a consistent measurable-disease denominator, explicit labelling of reconstructed and derived counts, the separation of single-arm from randomized evidence, and a statistical model appropriate for sparse binary data, complemented by extensive sensitivity analyses.

This review has limitations. The search covered MEDLINE/PubMed, Scopus, and Web of Science Core Collection, but Embase could not be searched because institutional access was unavailable; congress proceedings were hand-searched instead, but cohorts reported only at meetings or indexed only in Embase may have been missed. Title/abstract screening, data extraction, and risk-of-bias and GRADE assessments were performed by one reviewer (T.T.), with language-model assistance for data extraction; although every numeric value was verified manually against source documents, a second reviewer (K.S.) independently re-screened only the final full-text eligibility decisions, and duplicate independent extraction and appraisal were not performed, so extraction or judgement errors cannot be excluded. Four cohorts were available only as congress presentations or abstracts, and two estimates were reconstructed. Most cohorts were small subgroups, and only one trial was designed specifically to assess CNS efficacy. Response criteria and assessors differed, and TUXEDO-3 used local assessment. The zero estimate of τ^2^ is uncertain with eight cohorts and does not establish clinical homogeneity: the cohorts differed in target antigen, molecular selection, CNS status, response criteria, and imaging, so the pooled icORR is best read as an average across clinically diverse cohorts, and allowing for plausible heterogeneity widened its prediction interval to 9–69%. Small-study effects could not be assessed reliably, and selective reporting of favourable CNS subgroups cannot be excluded. Six of the eight cohorts were molecularly selected and most patients were heavily pretreated, so the estimates apply chiefly to previously treated, oncogene-driven NSCLC; evidence in molecularly unselected disease rests on two cohorts, and treatment-naïve disease is not represented. Safety data specific to patients with brain metastases were available for only three cohorts.

## 5. Conclusions

Deruxtecan-based ADCs produced intracranial responses in approximately one-third of patients with measurable brain metastases from previously treated, predominantly oncogene-driven NSCLC, with responses also observed in active disease, including in the only dedicated prospective CNS trial, and limited randomized data are compatible with delayed CNS progression compared with chemotherapy. Because certainty of evidence is very low to low, these findings should inform trial design rather than replace local therapy, and prospective trials with CNS-specific endpoints, measurable-disease denominators, and neurological outcomes are needed.

## Figures and Tables

**Table 1:** Characteristics of included trial cohorts.

Study [Ref]	Trial; Design; CNS Analysis	ADC; Dose (Target)	Population; Molecular Selection	CNS Status in Analyzed Subset	Measurable BM, n	Prior Brain RT or Local Therapy (Analyzed Subset)	Brain Imaging; Criteria; Assessor	Median Follow-Up; Report Type
Jänne et al. [18]	DESTINY-Lung02; randomized dose-blinded phase 2; post hoc	T-DXd 5.4 mg/kg Q3W (HER2)	Pretreated HER2-mutant NSCLC; selected	Stable (asymptomatic, no corticosteroids or anticonvulsants required); treated or untreated	14	8/14 prior RT (median 7.9 mo before entry)	MRI 5, CT 9; RECIST 1.1; BICR	11.5 mo (DESTINY-Lung02 [8]); peer-reviewed secondary analysis
Jänne et al. [18]	DESTINY-Lung01 + -Lung02; phase 2; post hoc	T-DXd 6.4 mg/kg Q3W (HER2)	Pretreated HER2-mutant NSCLC; selected	As above	30	14/30 prior RT (median 1.6 mo before entry)	MRI 20, CT 10; RECIST 1.1; BICR	13.1 and 11.8 mo (DESTINY-Lung01/02 [7,8]); peer-reviewed secondary analysis
Lisberg et al. [19]	TROPION-Lung05; single-arm phase 2; post hoc	Dato-DXd 6 mg/kg Q3W (TROP2)	NSCLC with actionable alterations after TT and Pt-CT; selected	Clinically stable	18	15/18 prior local therapy	NR; CNS RECIST; CNS BICR	NR; congress poster
Pons-Tostivint et al. [20]	TROPION-Lung01; phase 3 vs. docetaxel; post hoc	Dato-DXd 6 mg/kg Q3W (TROP2)	Pretreated NSCLC ± actionable alterations; unselected	Untreated or progressing after RT (restricted)	16 (docetaxel 11)	Untreated or progressing after RT; proportions NR	NR; CNS RECIST; CNS BICR	NR; congress oral
Yu et al. [11]	HERTHENA-Lung01; phase 2; exploratory CNS subgroup	HER3-DXd 5.6 mg/kg Q3W (HER3)	EGFR-mutant NSCLC after EGFR TKI and Pt-CT; selected	Non-irradiated (restricted)	7 R	None (non-irradiated by definition)	CT and/or MRI (6 of 10 responses on CT alone); CNS RECIST; CNS BICR	18.9 mo (median study duration); peer-reviewed
Mok et al. [21,24]	HERTHENA-Lung02; phase 3 vs. Pt-pemetrexed; CNS-PFS secondary endpoint	HER3-DXd 5.6 mg/kg Q3W (HER3)	EGFR-mutant NSCLC after 3rd-generation EGFR TKI; selected	Stable, not requiring corticosteroids or anticonvulsants	24 R (control 28)	37% prior brain RT	NR; CNS RECIST; CNS BICR	NR; congress oral
Li et al. [23]	DESTINY-Lung05; single-arm phase 2; CNS analysis	T-DXd 5.4 mg/kg Q3W (HER2)	Pretreated HER2-mutant NSCLC, China; selected	Pretreated, asymptomatic or neurologically stable (n = 30 with baseline CNS metastases)	10	NR	NR; CNS-modified RECIST 1.1; ICR	NR; congress abstract
Fuereder et al. [22]	TUXEDO-3 cohort 2; single-arm phase 2; dedicated CNS primary endpoint	HER3-DXd 5.6 mg/kg Q3W (HER3)	NSCLC, any molecular subtype (driver alteration 5/20); ECOG 0–2; unselected	Active: 7 untreated, 13 progressing after local therapy; no need for immediate local therapy (only dedicated CNS trial)	20 (≥1 lesion ≥10 mm on MRI)	13/20 progressing after local therapy	MRI; RANO-BM; local investigator	5.3 mo; peer-reviewed

All ADCs carry a deruxtecan (topoisomerase I inhibitor) payload. R, measurable-disease denominator with reconstructed responder count (derivation in File S6). Follow-up is that of the parent trial or analysis unless stated. NR, not reported in the available source. Abbreviations: BICR, blinded independent central review; BM, brain metastases; CT, computed tomography; ECOG, Eastern Cooperative Oncology Group; Dato-DXd, datopotamab deruxtecan; HER3-DXd, patritumab deruxtecan; ICR, independent central review; MRI, magnetic resonance imaging; Pt-CT, platinum-based chemotherapy; Q3W, every 3 weeks; RT, radiotherapy; T-DXd, trastuzumab deruxtecan; TKI, tyrosine kinase inhibitor; TT, targeted therapy.

**Table 2:** Intracranial outcomes by cohort.

Cohort [Ref]	icORR, Measurable BM, n/N (%; 95% CI)	icDCR, Measurable BM, n/N (%)	icORR, any CNS Lesion, n/N (%)	CNS Time-to-Event Outcomes
T-DXd 5.4 mg/kg [18]	7/14 (50%; 23–77); 3 CR	13/14 (93%) ^a^	NR	Median IC-DoR 9.5 mo
T-DXd 6.4 mg/kg [18]	9/30 (30%; 15–49)	22/30 (73%)	NR	Median IC-DoR 4.4 mo (2.9–10.2)
TROPION-Lung05 [19]	4/18 (22%; 6–48)	13/18 (72%)	NR	Median IC-PFS 6.9 mo (4.0–9.5), n = 53
TROPION-Lung01 [20]	6/16 (38%; 15–65) vs. docetaxel 0/11	88% reported (14/16 D) vs. 4/11	6/38 (16%) vs. 0/30	CNS-PFS (incl. death) 5.0 vs. 3.0 mo; HR 0.48 (0.23–0.98)
HERTHENA-Lung01 [11]	2/7 (29%; 4–71) R	NR	10/30 (33%); 9 CR	Median CNS-DoR 8.4 mo (5.8–9.2)
HERTHENA-Lung02 [21,24]	7/24 (29%; 13–51) R vs. 7/28 R	NR	20/105 (19%) vs. 11/95 (12%)	IC-PFS 5.4 vs. 4.2 mo; HR 0.75 (0.53–1.06) ^b^; IC-DoR 4.5 vs. 4.2 mo
DESTINY-Lung05 [23]	4/10 (40%; 12–74); 1 CR	10/10 (100%)	NR	Median CNS-DoR not evaluable
TUXEDO-3 [22]	6/20 (30%; 12–54); untreated 2/7, progressing 4/13	NR in sources reviewed	—	Median IC-PFS 4.8 mo (2.1–NR)

Observational (sensitivity analysis only): TRACER/HERTras [34], retrospective, investigator-assessed: icORR 20/27 (74%) in measurable brain metastases; 11/18 (61%) without local CNS therapy; 9/9 with local CNS therapy (derived). ^a^ As reported in the source. ^b^ Inclusion of death in the endpoint definition not reported in the available source. D, count derived arithmetically from reported percentages or best-overall-response categories; R, count reconstructed from lesion-classification rules (complete responses confined to non-target-only patients). Comparator values follow “vs”. 95% CIs are Clopper–Pearson and match published intervals where reported. Abbreviations: CR, complete response; DoR, duration of response; HR, hazard ratio; IC, intracranial; icDCR, intracranial disease control rate; icORR, intracranial objective response rate; NR, not reported; PFS, progression-free survival.

**Table 3:** GRADE summary of findings.

Outcome	Studies (Patients)	Effect (95% CI)	Risk of Bias	Inconsistency	Indirectness	Imprecision	Publication Bias	Certainty
icORR, measurable BM (single-arm)	8 cohorts (139)	32% (25–41)	Serious ^a^	Not serious	Not serious ^b^	Not serious	Undetected ^c^	Very low ⊕◯◯◯
icDCR, measurable BM (single-arm)	5 cohorts (88)	83% (69–92)	Serious ^a^	Not serious	Not serious ^b^	Not serious	Undetected ^c^	Very low ⊕◯◯◯
icORR, ADC vs. chemotherapy, measurable BM	2 RCTs (79)	Not pooled: RR 1.17 (0.48–2.85); 6/16 vs. 0/11	Serious ^d^	Not assessed ^e^	Not serious	Very serious ^f^	Undetected ^c^	Low ⊕⊕◯◯
CNS-PFS, ADC vs. chemotherapy	2 RCTs (268)	HR 0.48 (0.23–0.98) and 0.75 (0.53–1.06); not pooled (exploratory estimate in File S4)	Serious ^g^	Not serious	Not serious	Serious ^h^	Undetected ^c^	Low ⊕⊕◯◯

Single-arm proportions start at low certainty; randomized comparisons start at high. Judgements were made by one reviewer (T.T.). ^a^ Post hoc CNS subsets, congress-only data, and non-evaluable patients counted as non-responders (File S3, Table S1A). ^b^ Judged against the defined target population (adults with NSCLC brain metastases not requiring immediate local therapy); evidence does not extend to patients needing immediate local therapy. ^c^ Statistical assessment not possible with fewer than 10 cohorts; selective reporting of CNS subgroups cannot be excluded but no specific evidence of it was identified; not downgraded. ^d^ TROPION-Lung01 high risk and HERTHENA-Lung02 some concerns (File S3, Table S1B). ^e^ Results not pooled; different comparators may explain divergent estimates. ^f^ 20 responders in total; confidence intervals compatible with benefit and harm. ^g^ Post hoc CNS analysis with missing CNS imaging in TROPION-Lung01. ^h^ Only two trials; event counts not reported; the trial-level HR for HERTHENA-Lung02 includes no effect, and the exploratory pooled interval (File S4) spans a large to a small relative reduction. Abbreviations as in Table 2; RCT, randomized controlled trial; RR, risk ratio.

**Table 4:** Safety and corticosteroid data by cohort.

Cohort [Ref]	Safety Population (Level)	Grade ≥ 3 AEs	Adjudicated Drug-Related ILD (Grade 5)	Treatment-Related Deaths	Corticosteroids
T-DXd 5.4 mg/kg [8,18]	BM subgroup, n = 31 (CNS); DESTINY-Lung02 5.4 mg/kg, n = 101 (parent)	CNS: 65% (drug-related 39%); parent: drug-related 38.6%	CNS: 5/31 (16%); parent: 12.9% (grade ≥ 3 2.0%)	CNS: 1 (3%)	Excluded if ongoing corticosteroids required; use NR
T-DXd 6.4 mg/kg [7,8,18]	BM subgroup, n = 54 (CNS); DESTINY-Lung01, n = 91, and DESTINY-Lung02 6.4 mg/kg, n = 50 (parent)	CNS: 76% (drug-related 32/54); parent: drug-related 46% and 58.0%	CNS: 11/54 (20%); parent: 26% (2 deaths) and 28.0% (grade ≥ 3 2.0%)	CNS: 1 (2%)	As above
TROPION-Lung05 [9]	Parent, n = 137	Treatment-related 28.5%	3.6% (0.7%)	NR	NR
TROPION-Lung01 [10]	Parent, Dato-DXd vs. docetaxel	Treatment-related 25.6% vs. 42.1%	8.8% vs. 4.1%	NR	NR
HERTHENA-Lung01 [11]	Parent, n = 225	64.9% (any cause)	5.3% (0.4%)	4 (1.8%)	Excluded if required; RANO-BM corticosteroid data not routinely collected
HERTHENA-Lung02 [24]	Parent, n = 290 vs. 280	72.8% vs. 57.1% (treatment-related 57.9% vs. 46.1%)	5.2% (0.7%) vs. 0	NR	Excluded if required; use NR
DESTINY-Lung05 [23]	NR	NR	NR	NR	NR
TUXEDO-3 [22]	NSCLC cohort, n = 20 (CNS)	Grade 3–4 neutropenia 15%, febrile neutropenia 10% (any treatment-related AE 80%)	NR	0	NR

CNS, data from patients with brain metastases in the analyzed CNS cohort; parent, whole-trial safety population. Comparator values follow “vs”. ILD, interstitial lung disease/pneumonitis; NR, not reported in the available sources; other abbreviations as in Table 1 and Table 2.

## Data Availability

All data analyzed in this study were extracted from the published reports and presentations cited in the article. The extracted cohort-level dataset and the analysis code are publicly available in the Open Science Framework at https://doi.org/10.17605/OSF.IO/3RFQ7.

## Supplementary Materials

The following supporting information can be downloaded at: https://www.mdpi.com/article/10.3390/cancers18213552/s1, File S1: PRISMA 2020 checklist; File S2: full electronic search strategies for all sources; File S3: risk-of-bias and certainty assessments (Table S1A, MINORS appraisal of single-arm cohorts; Table S1B, RoB 2 appraisal of randomized CNS comparisons; GRADE rationale); File S4: sensitivity analyses (Table S2, sensitivity analyses of pooled intracranial objective response rate; Figure S1, leave-one-out analysis; Figure S2, exploratory pooled CNS-PFS hazard ratio); File S5: funnel plot and Egger regression test; File S6: derivation of reconstructed HERTHENA-Lung01 and HERTHENA-Lung02 counts and scenario analyses (Table S6a. Derivation of measurable-disease intracranial response counts; Table S6b. Scenario analyses for pooled intracranial objective response rate (GLMM); Table S6c. HERTHENA-Lung02 risk ratio for intracranial response in measurable disease (HER3-DXd vs platinum–pemetrexed), varying each arm by ±2 responders).

## Abbreviations

The following abbreviations are used in this manuscript:

ADC	antibody–drug conjugate
BICR	blinded independent central review
CI	confidence interval
CNS	central nervous system
CNS-PFS	CNS progression-free survival
Dato-DXd	datopotamab deruxtecan
GLMM	generalized linear mixed model
GRADE	Grading of Recommendations Assessment, Development and Evaluation
HER3-DXd	patritumab deruxtecan
HR	hazard ratio
icDCR	intracranial disease control rate
icORR	intracranial objective response rate
MINORS	Methodological Index for Non-Randomized Studies
NSCLC	non-small cell lung cancer
PRISMA	Preferred Reporting Items for Systematic Reviews and Meta-Analyses
RANO-BM	Response Assessment in Neuro-Oncology Brain Metastases
RoB 2	revised Cochrane risk-of-bias tool for randomized trials
T-DXd	trastuzumab deruxtecan
