# Supplementary material for: Intracranial Activity of Deruxtecan-Based Antibody–Drug Conjugates in Non-Small Cell Lung Cancer Brain Metastases: A Systematic Review and Meta-Analysis of Prospective Trial Cohorts
Source: Cancers (Basel). 2026 Nov 5;18(21):3552. doi: 10.3390/cancers18213552 (PMC13762147; doi:10.3390/cancers18213552)
Supplement: Supplementary file 1 [file cancers-18-03552-s001.zip › File_S1.pdf]

Intracranial Activity of Deruxtecan-Based Antibody–Drug Conjugates in Non-Small Cell Lung Cancer Brain Metastases: A Systematic Review and Meta-Analysis of Prospective Trial Cohorts

Tomasz Tykocki; Correspondence: ttykocki@gmail.com

File S1. PRISMA 2020 checklist

Completed against the submitted manuscript. Locations refer to manuscript section numbers, tables, figures, and supplementary files. Checklist items from Page et al., BMJ 2021;372:n71.

Section/topic	Item	Checklist item	Location where item is reported
**Title**	1	Identify the report as a systematic review.	Title
**Abstract**	2	See the PRISMA 2020 for Abstracts checklist.	Abstract
**Introduction**	3	Describe the rationale for the review in the context of existing knowledge.	Section 1, paragraphs 1–5
	4	Provide an explicit statement of the objective(s) or question(s) the review addresses.	Section 1, final paragraph
**Methods**	5	Specify the inclusion and exclusion criteria and how studies were grouped for the syntheses.	Sections 2.2 and 2.7
	6	Specify all databases, registers, websites, organisations, reference lists and other sources searched or consulted, with the date each was last searched.	Section 2.3; Figure 1; File S2
	7	Present the full search strategies for all databases, registers and websites, including filters and limits used.	File S2
	8	Specify the methods used to decide whether a study met the inclusion criteria, including reviewers, independence and automation tools.	Sections 2.1 and 2.4
	9	Specify the methods used to collect data from reports, including reviewers, independence, confirmation processes and automation tools.	Section 2.4 (single reviewer; large-language-model assistance; cross-checking against sources)
	10a	List and define all outcomes for which data were sought.	Section 2.5
	10b	List and define all other variables for which data were sought; describe assumptions about missing or unclear information.	Sections 2.4 and 2.5 (reconstructed/derived counts; non-evaluable patients)
	11	Specify the methods used to assess risk of bias in the included studies.	Section 2.6; File S3
	12	Specify for each outcome the effect measure(s) used.	Sections 2.5 and 2.7
	13a	Describe the processes used to decide which studies were eligible for each synthesis.	Sections 2.2 and 2.7
	13b	Describe any methods required to prepare the data for presentation or synthesis.	Sections 2.4, 2.5 and 2.7
	13c	Describe any methods used to tabulate or visually display results of individual studies and syntheses.	Section 2.7; Tables 1 and 2; Figures 2–4
	13d	Describe any methods used to synthesize results and provide a rationale; models, heterogeneity methods and software.	Section 2.7
	13e	Describe any methods used to explore possible causes	Section 2.7 (post hoc subgroups)

		of heterogeneity.	
	13f	Describe any sensitivity analyses conducted to assess robustness of the synthesized results.	Section 2.7; File S4
	14	Describe any methods used to assess risk of bias due to missing results (reporting biases).	Section 2.7 (small-study effects); File S5
	15	Describe any methods used to assess certainty (or confidence) in the body of evidence for an outcome.	Section 2.6
**Results**	16a	Describe the results of the search and selection process, ideally using a flow diagram.	Section 3.1; Figure 1
	16b	Cite studies that might appear to meet the inclusion criteria but were excluded, and explain why.	Section 3.2 (HORIZON-Lung/trastuzumab rezetecan; TRACER/HERTras); Figure 1 (exclusion reasons)
	17	Cite each included study and present its characteristics.	Section 3.2; Table 1
	18	Present assessments of risk of bias for each included study.	Section 3.6; File S3 (Tables S1A–B)
	19	For all outcomes, present summary statistics for each group and an effect estimate with precision for each study.	Table 2; Figures 2–4
	20a	For each synthesis, briefly summarise the characteristics and risk of bias among contributing studies.	Sections 3.2 and 3.6
	20b	Present results of all statistical syntheses conducted, with summary estimate, precision and heterogeneity.	Sections 3.3 and 3.5; Figures 2–4
	20c	Present results of all investigations of possible causes of heterogeneity.	Section 3.3; File S4 (Table S2)
	20d	Present results of all sensitivity analyses conducted.	Section 3.4; File S4
	21	Present assessments of risk of bias due to missing results for each synthesis assessed.	Section 3.6; Figure 5; File S5
	22	Present assessments of certainty (or confidence) in the body of evidence for each outcome assessed.	Section 3.6; Table 3; File S3
**Discussion**	23a	Provide a general interpretation of the results in the context of other evidence.	Sections 4.1–4.5
	23b	Discuss any limitations of the evidence included in the review.	Section 4.8
	23c	Discuss any limitations of the review processes used.	Section 4.8
	23d	Discuss implications of the results for practice, policy, and future research.	Sections 4.6 and 4.7; Section 5
**Other information**	24a	Provide registration information, including register name and number, or state that the review was not registered.	Section 2.1 (PROSPERO CRD420261516140; retrospective); Abstract
	24b	Indicate where the review protocol can be accessed, or state that a protocol was not prepared.	Section 2.1 (PROSPERO record)
	24c	Describe and explain any amendments to information provided at registration or in the protocol.	Section 2.7, final paragraph
	25	Describe sources of financial or non-financial support for the review, and the role of the funders.	Funding statement
	26	Declare any competing interests of review authors.	Conflicts of Interest statement
	27	Report which data, code and other materials are publicly available and where.	Data Availability Statement

PRISMA 2020 for Abstracts checklist

Topic	Checklist item	Reported in Abstract
Title	Identify the report as a systematic review.	Yes
Objectives	Provide an explicit statement of the main objective(s).	Yes
Eligibility criteria	Specify the inclusion and exclusion criteria.	Yes (prospective trials of ADC monotherapy in NSCLC reporting CNS outcomes)
Information sources	Specify the information sources and the date each was last searched.	Yes
Risk of bias	Specify the methods used to assess risk of bias.	Partly (GRADE named; MINORS/RoB 2 in Section 2.6)
Synthesis of results	Specify the methods used to present and synthesize results.	Yes
Included studies	Give the number of included studies and participants.	Yes
Synthesis of results	Present results for main outcomes, with summary estimate and CI.	Yes
Limitations of evidence	Provide a brief summary of the limitations of the evidence.	Yes (certainty very low to low)
Interpretation	Provide a general interpretation of the results and important implications.	Yes
Funding	Specify the primary source of funding for the review.	No (no funding; stated in back matter)
Registration	Provide the register name and registration number.	Yes (PROSPERO CRD420261516140)
