# Supplementary material for: Intracranial Activity of Deruxtecan-Based Antibody–Drug Conjugates in Non-Small Cell Lung Cancer Brain Metastases: A Systematic Review and Meta-Analysis of Prospective Trial Cohorts
Source: Cancers (Basel). 2026 Nov 5;18(21):3552. doi: 10.3390/cancers18213552 (PMC13762147; doi:10.3390/cancers18213552)
Supplement: Supplementary file 1 [file cancers-18-03552-s001.zip › File_S2.pdf]

Cancers

Intracranial Activity of Deruxtecan-Based Antibody–Drug Conjugates in Non-Small Cell Lung Cancer Brain Metastases: A Systematic Review and Meta-Analysis of Prospective Trial Cohorts

Tomasz Tykocki

Department of Paediatric Neurosurgery, Children's Hospital named after prof. dr med. Jan Bogdanowicz, Warsaw, Poland; Faculty of Medicine, Lazarski University, Warsaw, Poland

Corresponding author: ttykocki@gmail.com

File S2. Full electronic search strategies

Intracranial activity of antibody-drug conjugates in NSCLC brain metastases

Search date: 21 September 2026
No publication-date or language restrictions were applied at the search stage.
Embase was not searched because institutional access was unavailable.

1. MEDLINE via PubMed

Platform/database: PubMed

Note: CNS-focused search used to identify publications with intracranial outcomes.

```
(
  "Carcinoma, Non-Small-Cell Lung"[Mesh]
  OR NSCLC[tiab]
  OR "non-small cell lung cancer"[tiab]
  OR "non-small-cell lung cancer"[tiab]
  OR "non small cell lung cancer"[tiab]
)
AND
(
  "Immunoconjugates"[Mesh]
  OR "antibody-drug conjugate"[tiab]
  OR "antibody-drug conjugates"[tiab]
  OR "antibody drug conjugate"[tiab]
  OR "antibody drug conjugates"[tiab]
  OR deruxtecan[tiab]
  OR "trastuzumab deruxtecan"[tiab]
  OR "T-DXd"[tiab]
  OR "DS-8201"[tiab]
  OR "DS-8201a"[tiab]
  OR "datopotamab deruxtecan"[tiab]
  OR "Dato-DXd"[tiab]
  OR "DS-1062"[tiab]
  OR "DS-1062a"[tiab]
  OR "patritumab deruxtecan"[tiab]
  OR "HER3-DXd"[tiab]
  OR "U3-1402"[tiab]
  OR "trastuzumab rezetecan"[tiab]
  OR "SHR-A1811"[tiab]
  OR "trastuzumab emtansine"[tiab]
```

```
    OR "T-DM1"[tiab]
    OR "sacituzumab govitecan"[tiab]
    OR "sacituzumab tirumotecan"[tiab]
    OR "SKB264"[tiab]
    OR "MK-2870"[tiab]
    OR "telisotuzumab vedotin"[tiab]
    OR "Teliso-V"[tiab]
    OR "ABBV-399"[tiab]
    OR "tusamitamab ravtansine"[tiab]
    OR "SAR408701"[tiab]
    OR "BL-B01D1"[tiab]
    OR "izalontamab brengitecan"[tiab]
  )
  AND
  (
    "Brain Neoplasms/secondary"[Mesh]
    OR "brain metastasis"[tiab]
    OR "brain metastases"[tiab]
    OR "brain metastas*"[tiab]
    OR "cerebral metastasis"[tiab]
    OR "cerebral metastases"[tiab]
    OR "cerebral metastas*"[tiab]
    OR intracranial[tiab]
    OR "CNS metastasis"[tiab]
    OR "CNS metastases"[tiab]
    OR "CNS metastas*"[tiab]
    OR "central nervous system metastasis"[tiab]
    OR "central nervous system metastases"[tiab]
    OR "central nervous system metastas*"[tiab]
  )
```

2. Scopus

Platform/database: Scopus Advanced Search

```
  TITLE-ABS-KEY(
    ("non-small cell lung cancer"
     OR "non-small-cell lung cancer"
     OR "non small cell lung cancer"
     OR NSCLC)
    AND
    ("antibody-drug conjugate*"
     OR "antibody drug conjugate*"
     OR deruxtecan
     OR "trastuzumab deruxtecan"
     OR "T-DXd"
     OR "DS-8201"
     OR "DS-8201a"
     OR "datopotamab deruxtecan"
     OR "Dato-DXd"
     OR "DS-1062"
     OR "DS-1062a"
     OR "patritumab deruxtecan"
     OR "HER3-DXd"
     OR "U3-1402"
     OR "trastuzumab rezetecan"
     OR "SHR-A1811"
     OR "trastuzumab emtansine"
     OR "T-DM1"
     OR "sacituzumab govitecan"
     OR "sacituzumab tirumotecan"
     OR "SKB264"
```

```
   OR "MK-2870"
   OR "telisotuzumab vedotin"
   OR "Teliso-V"
   OR "ABBV-399"
   OR "tusamitamab ravtansine"
   OR "SAR408701"
   OR "BL-B01D1"
   OR "izalontamab brengitecan")
  AND
  ("brain metastas*"
   OR "cerebral metastas*"
   OR intracranial
   OR "CNS metastas*"
   OR "central nervous system metastas*")
)
```

3. Web of Science Core Collection

Platform/database: Web of Science Advanced Search / Query Builder

Note: If using Fielded Search with Topic selected, omit the leading TS= and paste only the expression inside the parentheses.

```
TS=(
  ("non-small cell lung cancer"
   OR "non-small-cell lung cancer"
   OR "non small cell lung cancer"
   OR NSCLC)
  AND
  ("antibody-drug conjugate*"
   OR "antibody drug conjugate*"
   OR deruxtecan
   OR "trastuzumab deruxtecan"
   OR "T-DXd"
   OR "DS-8201"
   OR "DS-8201a"
   OR "datopotamab deruxtecan"
   OR "Dato-DXd"
   OR "DS-1062"
   OR "DS-1062a"
   OR "patritumab deruxtecan"
   OR "HER3-DXd"
   OR "U3-1402"
   OR "trastuzumab rezetecan"
   OR "SHR-A1811"
   OR "trastuzumab emtansine"
   OR "T-DM1"
   OR "sacituzumab govitecan"
   OR "sacituzumab tirumotecan"
   OR "SKB264"
   OR "MK-2870"
   OR "telisotuzumab vedotin"
   OR "Teliso-V"
   OR "ABBV-399"
   OR "tusamitamab ravtansine"
   OR "SAR408701"
   OR "BL-B01D1"
   OR "izalontamab brengitecan")
  AND
  ("brain metastas*"
   OR "cerebral metastas*"
   OR intracranial
```

```
   OR "CNS metastas*"
   OR "central nervous system metastas*")
)
```

4. Cochrane CENTRAL

Platform/database: Cochrane Library - CENTRAL

```
#1 MeSH descriptor: [Carcinoma, Non-Small-Cell Lung] explode all trees
#2 ("non-small cell lung cancer" OR "non-small-cell lung cancer" OR "non small cell lung
cancer" OR NSCLC):ti,ab,kw
#3 #1 OR #2

#4 MeSH descriptor: [Immunoconjugates] explode all trees
#5 ("antibody-drug conjugate*" OR "antibody drug conjugate*" OR deruxtecan
    OR "trastuzumab deruxtecan" OR "T-DXd" OR "DS-8201" OR "DS-8201a"
    OR "datopotamab deruxtecan" OR "Dato-DXd" OR "DS-1062" OR "DS-1062a"
    OR "patritumab deruxtecan" OR "HER3-DXd" OR "U3-1402"
    OR "trastuzumab rezetecan" OR "SHR-A1811"
    OR "trastuzumab emtansine" OR "T-DM1"
    OR "sacituzumab govitecan" OR "sacituzumab tirumotecan"
    OR "SKB264" OR "MK-2870"
    OR "telisotuzumab vedotin" OR "Teliso-V" OR "ABBV-399"
    OR "tusamitamab ravtansine" OR "SAR408701"
    OR "BL-B01D1" OR "izalontamab brengitecan"):ti,ab,kw
#6 #4 OR #5

#7 MeSH descriptor: [Brain Neoplasms] explode all trees
#8 ("brain metastas*" OR "cerebral metastas*" OR intracranial
    OR "CNS metastas*" OR "central nervous system metastas*"):ti,ab,kw
#9 #7 OR #8

#10 #3 AND #6 AND #9
```

5. ClinicalTrials.gov

Platform/database: ClinicalTrials.gov

Note: Registry searching was supplementary and used both generic ADC terms and agent/trial-specific names.

```
Condition/disease:
"non-small cell lung cancer" OR NSCLC

Other terms:
("antibody-drug conjugate" OR ADC OR deruxtecan
 OR "trastuzumab deruxtecan" OR "T-DXd"
 OR "datopotamab deruxtecan" OR "Dato-DXd"
 OR "patritumab deruxtecan" OR "HER3-DXd"
 OR "trastuzumab rezetecan" OR "SHR-A1811"
 OR "sacituzumab govitecan" OR "sacituzumab tirumotecan"
 OR "telisotuzumab vedotin" OR "BL-B01D1"
 OR "izalontamab brengitecan")
AND
("brain metastases" OR intracranial OR CNS)
```

6. Europe PMC / PubMed Central

Platform/database: Europe PMC

Note: Supplementary search source; overlaps substantially with PubMed/MEDLINE and PMC.

```
TITLE_ABS:
(
 ("non-small cell lung cancer" OR "non-small-cell lung cancer" OR NSCLC)
 AND
 ("antibody-drug conjugate" OR "antibody drug conjugate" OR deruxtecan
  OR "trastuzumab deruxtecan" OR "T-DXd"
  OR "datopotamab deruxtecan" OR "Dato-DXd"
  OR "patritumab deruxtecan" OR "HER3-DXd"
  OR "trastuzumab rezetecan" OR "SHR-A1811"
  OR "sacituzumab govitecan" OR "sacituzumab tirumotecan"
  OR "telisotuzumab vedotin" OR "BL-B01D1"
  OR "izalontamab brengitecan")
 AND
 ("brain metastas*" OR "cerebral metastas*" OR intracranial
  OR "CNS metastas*" OR "central nervous system metastas*")
)
```

7. Conference proceedings

Platform/database: ASCO, ESMO, WCLC / Journal of Thoracic Oncology

Note: Congress searching was supplementary and included generic concept searches plus trial-name searching.

```
Conference sources searched: ASCO, ESMO, and WCLC/JTO.

Core search concept:
("non-small cell lung cancer" OR NSCLC)
AND
("antibody-drug conjugate" OR ADC OR deruxtecan
 OR "trastuzumab deruxtecan" OR "T-DXd"
 OR "datopotamab deruxtecan" OR "Dato-DXd"
 OR "patritumab deruxtecan" OR "HER3-DXd"
 OR "trastuzumab rezetecan" OR "SHR-A1811"
 OR "sacituzumab govitecan" OR "sacituzumab tirumotecan"
 OR "telisotuzumab vedotin" OR "BL-B01D1")
AND
("brain metastases" OR intracranial OR CNS)

Agent-specific trial names were also searched where relevant:
DESTINY-Lung01, DESTINY-Lung02, DESTINY-Lung05,
TROPION-Lung01, TROPION-Lung05,
HERTHENA-Lung01, HERTHENA-Lung02, TUXEDO-3, HORIZON-Lung.
```

Reporting note. The full search strategies should be reported exactly as executed in the final manuscript or supplement. If any syntax was modified in the live database interface, the final saved search history should replace the corresponding draft string above.
