# Supplementary material for: Intracranial Activity of Deruxtecan-Based Antibody–Drug Conjugates in Non-Small Cell Lung Cancer Brain Metastases: A Systematic Review and Meta-Analysis of Prospective Trial Cohorts
Source: Cancers (Basel). 2026 Nov 5;18(21):3552. doi: 10.3390/cancers18213552 (PMC13762147; doi:10.3390/cancers18213552)
Supplement: Supplementary file 1 [file cancers-18-03552-s001.zip › File_S3.pdf]

Cancers

Intracranial Activity of Deruxtecan-Based Antibody–Drug Conjugates in Non-Small Cell Lung Cancer Brain Metastases: A Systematic Review and Meta-Analysis of Prospective Trial Cohorts

Tomasz Tykocki
Department of Paediatric Neurosurgery, Children's Hospital named after prof. dr med. Jan Bogdanowicz, Warsaw, Poland; Faculty of Medicine, Lazarski University, Warsaw, Poland
Corresponding author: ttykocki@gmail.com

File S3. Risk of bias and certainty of evidence (GRADE)

Methods

Single-arm intracranial estimates were appraised with the eight non-comparative items of the Methodological Index for Non-Randomized Studies (MINORS). Randomized CNS comparisons were appraised with the revised Cochrane risk-of-bias tool (RoB 2), separately for intracranial response and CNS progression-free survival. Certainty of evidence was rated per outcome using GRADE against the target population of adults with advanced NSCLC and brain metastases not requiring immediate local therapy. Non-comparative proportions started at low certainty and randomized comparisons at high certainty. All assessments were performed by a single reviewer.

Certainty of evidence

Intracranial objective response rate (single-arm; 8 cohorts, 139 patients): very low certainty. Pooled icORR was 32% (95% CI, 25–41%). Certainty started low because the estimate is non-comparative and was downgraded one level for risk of bias: most CNS analyses were post hoc subsets, two counts were reconstructed, four cohorts were available only as congress abstracts or presentations, and non-evaluable patients were counted as non-responders. Inconsistency was not serious ($I^2 = 0\%$; prediction interval, 24–43%), indirectness was not serious relative to the defined population, and imprecision was not serious. Small-study effects could not be assessed reliably (File S5).

Intracranial disease control rate (single-arm; 5 cohorts, 88 patients): very low certainty. Pooled icDCR was 83% (69–92%), downgraded for risk of bias for the same reasons; the estimate was model dependent (inverse-variance 79%).

Intracranial response, ADC versus chemotherapy (2 randomized trials, 79 patients with measurable disease): low certainty. Results were not pooled (TROPION-Lung01, 6/16 vs 0/11; HERTHENA-Lung02, risk ratio 1.17 [0.48–2.85]). Downgraded one level for risk of bias (high risk in TROPION-Lung01; some concerns in HERTHENA-Lung02) and two levels for imprecision (20 responders in total; intervals compatible with benefit and harm).

CNS progression-free survival, ADC versus chemotherapy (2 randomized trials, 268 patients): low certainty. Trial-level HRs were 0.48 (0.23–0.98) and 0.75 (0.53–1.06); exploratory pooled HR 0.69 (0.50–0.94). Downgraded one level for risk of bias (post hoc CNS analysis with missing CNS imaging in TROPION-Lung01) and one level for imprecision (two trials, event counts not reported, trial-level interval including no effect).

Because GRADE was developed for comparative effects, its application to absolute single-arm proportions is less established; the ratings for single-arm outcomes should be read together with the risk-of-bias appraisal and the narrative above.

The GRADE summary-of-findings table is presented as Table 3 in the main article.

Table S1. Risk-of-bias assessment

A. MINORS, non-comparative items (0 = not reported, 1 = reported but inadequate, 2 = reported and adequate; NA = not assessable from available sources). Items: 1 clearly stated aim; 2 consecutive patients; 3 prospective data collection; 4 appropriate endpoint; 5 unbiased endpoint assessment; 6 appropriate follow-up; 7 loss to follow-up <5%; 8 prospective study-size calculation. Totals are given over verified items only and should not be interpreted as summary quality scores.

**Cohort**	**1**	**2**	**3**	**4**	**5**	**6**	**7**	**8**	**Verified items (score / max)**	**Key concerns**
T-DXd 5.4 mg/kg	2	NA	2	2	2	NA	NA	0	8 / 10	Post hoc CNS analysis
T-DXd 6.4 mg/kg	2	NA	2	2	2	NA	1	0	9 / 12	Post hoc; 6.7% missing
TROPION-Lung05	2	NA	2	2	2	NA	1	0	9 / 12	Post hoc; 3/18 not evaluable
TROPION-Lung01	2	NA	2	2	2	NA	1	0	9 / 12	Post hoc; 2/16 not evaluable
HERTHENA-Lung01	1	NA	2	1	2	NA	NA	0	6 / 10	CNS analysis not primary aim; CT-only responses
HERTHENA-Lung02 (ADC arm)	1	NA	2	2	2	2	1	0	10 / 14	icORR not a listed endpoint; 6/105 not evaluable
TUXEDO-3	2	NA	2	2	1	NA	NA	2	9 / 10	Local unblinded assessment
DESTINY-Lung05	2	NA	2	2	2	NA	NA	0	8 / 10	Abstract only; CNS analysis exploratory

Reconstructed counts (HERTHENA-Lung01, HERTHENA-Lung02) are extraction uncertainties of this review and are handled in sensitivity analyses, not scored as study-level bias.

B. RoB 2 for randomized CNS comparisons (D1 randomization; D2 deviations from intended interventions; D3 missing outcome data; D4 outcome measurement; D5 selection of reported result).

**Trial; result**	**D1 Randomization**	**D2 Deviations**	**D3 Missing data**	**D4 Measurement**	**D5 Selection of result**	**Overall**
TROPION-Lung01; icORR, measurable untreated/progressing BM	Low: randomized; subset defined by baseline BICR findings	Some concerns: no information on deviations	High: no adequate CNS assessment in 2/16 vs 5/11; reasons not reported, imbalance	Low: CNS BICR, CNS RECIST	Some concerns: one of several post hoc CNS subsets	High
TROPION-Lung01; CNS-PFS (incl. death)	Low	Some concerns: no information	Some concerns: investigators note censoring driven by missing CNS imaging	Low: CNS BICR	Some concerns: post hoc subset	Some concerns
HERTHENA-Lung02; icORR, measurable BM	Low: stratified by stable BM	Some concerns: no information	Some concerns: measurable-subset missingness not reported (all-lesion NE 6/105 vs 11/95)	Low: CNS BICR	Some concerns: icORR not a listed endpoint	Some concerns
HERTHENA-Lung02; intracranial PFS	Low	Some concerns: no information	Some concerns: censoring pattern not reported	Low: CNS BICR	Low: prespecified secondary endpoint	Some concerns

Judgements were made by a single reviewer from publicly available reports.
