# Supplementary material for: Intracranial Activity of Deruxtecan-Based Antibody–Drug Conjugates in Non-Small Cell Lung Cancer Brain Metastases: A Systematic Review and Meta-Analysis of Prospective Trial Cohorts
Source: Cancers (Basel). 2026 Nov 5;18(21):3552. doi: 10.3390/cancers18213552 (PMC13762147; doi:10.3390/cancers18213552)
Supplement: Supplementary file 1 [file cancers-18-03552-s001.zip › File_S4.pdf]

Cancers

Intracranial Activity of Deruxtecan-Based Antibody–Drug Conjugates in Non-Small Cell Lung Cancer Brain Metastases: A Systematic Review and Meta-Analysis of Prospective Trial Cohorts

Tomasz Tykocki
Department of Paediatric Neurosurgery, Children's Hospital named after prof. dr med. Jan Bogdanowicz, Warsaw, Poland; Faculty of Medicine, Lazarski University, Warsaw, Poland
Corresponding author: ttykocki@gmail.com

File S4. Sensitivity analyses

Methods

All sensitivity analyses were post hoc. The primary model was a random-intercept logistic regression (binomial–normal GLMM, maximum likelihood). Alternative models were inverse-variance random-effects meta-analysis of logit proportions with REML estimation of τ^2, with unmodified and modified Hartung–Knapp–Sidik–Jonkman adjustment. Robustness was examined by leave-one-out analysis, exclusion of reconstructed response counts (HERTHENA-Lung01, HERTHENA-Lung02), restriction to peer-reviewed reports, restriction to independent central review (omitting TUXEDO-3), and addition of observational evidence from the TRACER/HERTras real-world cohort, first restricted to patients without local CNS therapy during or immediately before trastuzumab deruxtecan (11/18) and then including all patients with measurable metastases (20/27).

Additional sensitivity analyses were performed at peer review. Because τ^2 estimated as zero from eight small cohorts does not establish homogeneity, the GLMM was refitted with τ^2 fixed at the upper limit of its Q-profile 95% confidence interval, with the 95% prediction interval recalculated (t distribution, $k - 2$ degrees of freedom), and with τ^2 fixed at 0.10 for illustration; Bayesian binomial–normal models with a flat prior on the mean logit and half-normal priors on τ (scale 0.5 and 1.0) were fitted by grid integration. For intracranial disease control, the analysis was repeated after excluding DESTINY-Lung05 and with a half-normal(0.5) prior on τ. Code and data: https://doi.org/10.17605/OSF.IO/3RFQ7.

Results

Across prospective-only analyses, pooled icORR ranged from 30% to 34% (Table S2; Figure S1). No single cohort changed the estimate by more than 2 percentage points. Excluding reconstructed counts gave 33% (95% CI, 25–43%), and restricting to peer-reviewed reports gave 34% (24–46%). Adding TRACER/HERTras patients without local CNS therapy increased the estimate to 36% (28–44%) with little heterogeneity ($I^2 = 7\%$). Adding the whole TRACER/HERTras measurable cohort increased it to 38% (27–50%) and introduced substantial heterogeneity ($I^2 = 54\%$; $P = 0.03$), consistent with confounding by concomitant local therapy, since all 9 locally treated patients responded.

For intracranial disease control, the GLMM gave 83% (95% CI, 69–92%) and the inverse-variance model 79% (62–90%); the difference reflects the DESTINY-Lung05 cohort with 10 of 10 patients achieving disease control. Excluding DESTINY-Lung05 gave 79% (69–87%), and the Bayesian model 82% (72–88%). Of the 72 patients with disease control, 30 had an objective response and 42 had stable disease. For CNS progression-free survival, the exploratory common-effect pooled HR was 0.69 (0.50–0.94; $I^2 = 16\%$) and the DerSimonian–Laird random-effects HR 0.68 (0.47–0.98) (Figure S2); this estimate is exploratory because the two trials differ in ADC, comparator, CNS population, and endpoint definition.

The Q-profile 95% confidence interval for τ^2 was 0 to 0.31. With τ^2 fixed at 0.31, pooled icORR was 32% (95% CI, 22–45%) with a prediction interval of 9–69%; with τ^2 fixed at 0.10, 32% (24–42%) with a prediction interval of 16–55%. Bayesian models gave 32% (24–42%) with both prior scales (posterior median τ 0.17 and 0.19). The point estimate was therefore stable, whereas the range of effects expected in a new cohort was considerably wider than under the zero-heterogeneity model (Table S2).

Table S2. Sensitivity analyses of pooled intracranial objective response rate

Analysis	Cohorts	Patients	Pooled icORR (95% CI)	Prespecified
Primary: GLMM, prospective cohorts	8	139	32% (25–41)	No a
Inverse-variance, REML, Hartung–Knapp (unmodified)	8	139	33% (26–40)	No
Inverse-variance, REML, Hartung–Knapp (modified)	8	139	33% (24–43)	No
Omit T-DXd 5.4 mg/kg	7	125	30% (23–39)	No
Omit T-DXd 6.4 mg/kg	7	109	33% (25–42)	No
Omit DESTINY-Lung05	7	129	32% (24–40)	No
Omit TROPION-Lung05	7	121	34% (26–43)	No
Omit TROPION-Lung01	7	123	32% (24–40)	No
Omit HERTHENA-Lung01	7	132	33% (25–41)	No
Omit HERTHENA-Lung02	7	115	33% (25–42)	No
Omit TUXEDO-3 (central review only)	7	119	33% (25–42)	No
Exclude reconstructed counts	6	108	33% (25–43)	No
Peer-reviewed reports only	4	71	34% (24–46)	No
+ TRACER/HERTras, no local CNS therapy (11/18)	9	157	36% (28–44); $I^2 = 7\%$	No
+ TRACER/HERTras, all measurable (20/27; confounded by local therapy)	9	166	38% (27–50); $I^2 = 54\%$	No
Descriptive: cohorts with eligibility restricted to untreated/progressing BM	3	43	33% (20–48)	No
Descriptive: cohorts with mixed or unspecified eligibility	5	96	32% (24–42)	No
GLMM, τ^2 fixed at Q-profile upper limit (0.31); PI 9–69%	8	139	32% (22–45)	No
GLMM, τ^2 fixed at 0.10; PI 16–55%	8	139	32% (24–42)	No
Bayesian binomial–normal, half-normal(0.5) prior on τ	8	139	32% (24–42)	No
Bayesian binomial–normal, half-normal(1.0) prior on τ	8	139	32% (24–42)	No
HERTHENA counts, lower scenario (1/7; 5/24)	8	139	30% (23–38)	No
HERTHENA counts, higher scenario (3/7; 9/24)	8	139	35% (27–43)	No
HERTHENA counts, extreme lower (0/7; 4/24)	8	139	29% (22–37)	No
HERTHENA counts, extreme higher (4/7; 12/24)	8	139	37% (30–46)	No

a No analysis plan was documented in advance. The eligibility comparison ($Chi^2 < 0.01$, df = 1, P = 0.99) does not separate active from stable disease and does not establish similar activity; by target antigen: $Chi^2 = 0.87$, df = 2, P = 0.65. CNS-PFS random-effects (DerSimonian–Laird) HR 0.68 (0.47–0.98); $\tau^2 = 0.02$; $I^2 = 16\%$.

PI, 95% prediction interval. Rows added at peer review: heterogeneity-permitting models and HERTHENA scenario analyses (derivation in File S6).

Figure S1. Leave-one-out analysis of pooled intracranial objective response rate (GLMM). Diamond, primary estimate; squares, estimates after omitting each cohort; dotted line, primary estimate.

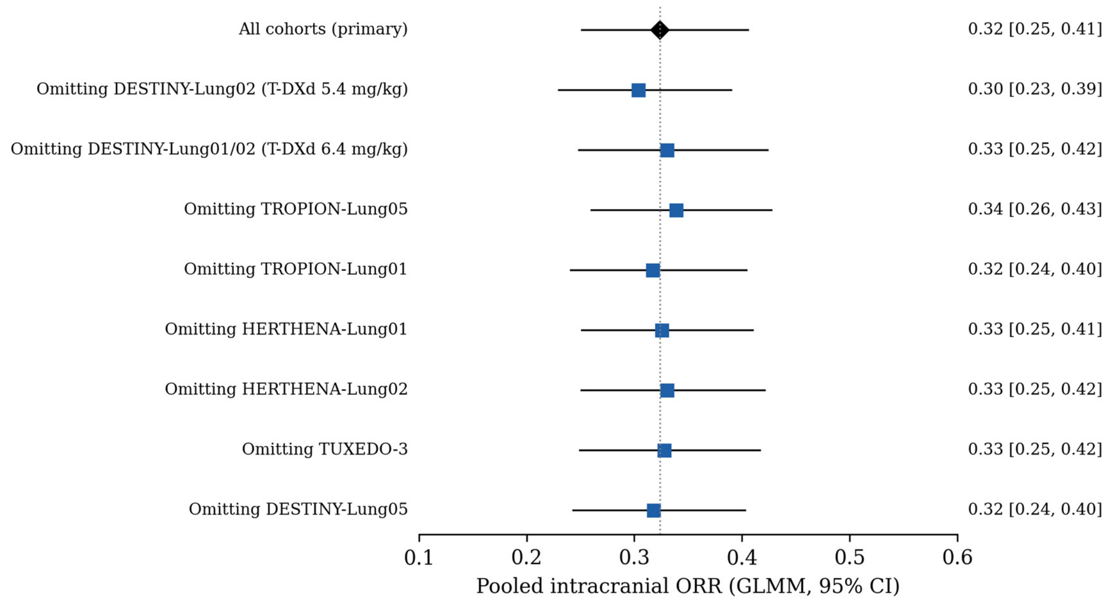

Study or Subgroup	ADC<br>Events/Total	Control<br>Events/Total	Weight	
**A. Intracranial ORR, measurable brain metastases (risk ratio; not pooled)**				**Risk ratio [95% CI]**
Pons-Tostivint et al. (TROPION-Lung01) vs docetaxel	6/16	0/11		Not estimable†
Mok et al. (HERTHENA-Lung02) vs platinum–pemetrexed *	7/24	7/28		1.17 [0.48, 2.85]
**B. Intracranial ORR, any CNS lesion (risk ratio; not pooled)**				**Risk ratio [95% CI]**
Pons-Tostivint et al. (TROPION-Lung01) vs docetaxel	6/38	0/30		Not estimable†
Mok et al. (HERTHENA-Lung02) vs platinum–pemetrexed	20/105	11/95		1.65 [0.83, 3.25]
**C. CNS progression-free survival (hazard ratio; exploratory IV common-effect pooling)**				**Hazard ratio, IV [95% CI]**
Pons-Tostivint et al. (TROPION-Lung01) vs docetaxel (n = 38 vs 30)			18.6%	0.48 [0.23, 0.98]
Mok et al. (HERTHENA-Lung02) vs platinum–pemetrexed (n = 105 vs 95)			81.4%	0.75 [0.53, 1.06]
**Exploratory pooled (95% CI)**			**100.0%**	**0.69 [0.50, 0.94]**

Heterogeneity: Chi² = 1.19, df = 1 (P = 0.28); I² = 16%. Test for overall effect: Z = 2.32 (P = 0.02)

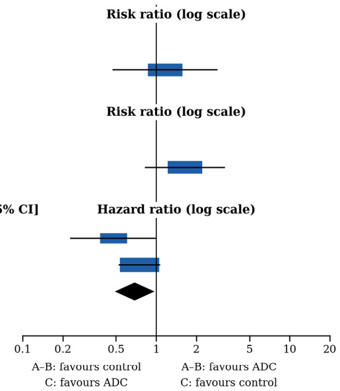

Panels A–B: study-level risk ratios (Wald 95% CI); not pooled because of two trials with different comparators and a zero-event control arm.

† Risk ratio not estimable (0 control events; no continuity correction applied). * Reconstructed counts (7/24 vs 7/28).

Panel C: exploratory inverse-variance common-effect pooling; trials differ in ADC, comparator and population, so a common effect cannot be assumed.

TROPION-Lung01: untreated or progressing brain metastases, endpoint includes death; HERTHENA-Lung02: ≥1 CNS lesion, death inclusion not confirmed.

Figure S2. Randomized CNS outcomes with the exploratory inverse-variance common-effect pooled CNS progression-free survival hazard ratio (0.69, 95% CI 0.50–0.94; I² = 16%; DerSimonian–Laird random-effects 0.68, 0.47–0.98). Panels A and B: study-level risk ratios, not pooled. The trials differ in ADC, comparator, CNS population, and endpoint definition; the pooled estimate is exploratory and is not presented in the main text.
