# Supplementary material for: Intracranial Activity of Deruxtecan-Based Antibody–Drug Conjugates in Non-Small Cell Lung Cancer Brain Metastases: A Systematic Review and Meta-Analysis of Prospective Trial Cohorts
Source: Cancers (Basel). 2026 Nov 5;18(21):3552. doi: 10.3390/cancers18213552 (PMC13762147; doi:10.3390/cancers18213552)
Supplement: Supplementary file 1 [file cancers-18-03552-s001.zip › File_S5.pdf]

Cancers

Intracranial Activity of Deruxtecan-Based Antibody–Drug Conjugates in Non-Small Cell Lung Cancer Brain Metastases: A Systematic Review and Meta-Analysis of Prospective Trial Cohorts

Tomasz Tykocki
Department of Paediatric Neurosurgery, Children's Hospital named after prof. dr med. Jan Bogdanowicz, Warsaw, Poland; Faculty of Medicine, Lazarski University, Warsaw, Poland
Corresponding author: ttykocki@gmail.com

File S5. Funnel plot and Egger regression test

Methods

Small-study effects for the primary outcome were explored with a funnel plot of logit-transformed intracranial objective response rates against their standard errors, with pseudo-95% confidence limits around the pooled estimate. Funnel-plot asymmetry was tested with Egger regression (standardized effect regressed on precision; intercept tested with a t distribution, k − 2 degrees of freedom). Because the logit of a proportion and its standard error are mathematically correlated, a complementary regression of the logit proportion on the inverse of sample size (weighted by inverse variance) was also performed. These analyses were post hoc and exploratory: fewer than 10 cohorts were available, below the minimum generally recommended for asymmetry tests, and funnel plots are known to perform poorly in meta-analyses of proportions.

Results

The funnel plot of eight cohorts showed no visually marked asymmetry (Figure 5 in the main article). The Egger intercept was 0.44 (95% CI, -2.88 to 3.76; t = 0.32, df = 6, P = 0.76). The sample-size-based regression also gave no evidence of small-study effects (slope P = 0.43).

Interpretation

A non-significant test with eight cohorts has very low power and does not exclude small-study effects or publication bias. Funnel-plot asymmetry, had it been present, would not have been specific to publication bias, because heterogeneity in populations, response criteria, and assessment methods can also produce asymmetry. Selective reporting of favourable CNS

subgroups from larger trials, which is the more plausible mechanism of bias in this field, cannot be detected by these methods. Publication bias was therefore not used to downgrade certainty, and its assessment is considered inconclusive.
