# Supplementary material for: Intracranial Activity of Deruxtecan-Based Antibody–Drug Conjugates in Non-Small Cell Lung Cancer Brain Metastases: A Systematic Review and Meta-Analysis of Prospective Trial Cohorts
Source: Cancers (Basel). 2026 Nov 5;18(21):3552. doi: 10.3390/cancers18213552 (PMC13762147; doi:10.3390/cancers18213552)
Supplement: Supplementary file 1 [file cancers-18-03552-s001.zip › File_S6.pdf]

Cancers
Intracranial Activity of Deruxtecan-Based Antibody–Drug Conjugates in Non-Small Cell Lung Cancer Brain Metastases: A Systematic Review and Meta-Analysis of Prospective Trial Cohorts
Tomasz Tykocki
Department of Paediatric Neurosurgery, Children's Hospital named after prof. dr med. Jan Bogdanowicz, Warsaw, Poland; Faculty of Medicine, Lazarski University, Warsaw, Poland
Corresponding author: ttykocki@gmail.com

File S6. Derivation of reconstructed HERTHENA-Lung01 and HERTHENA-Lung02 counts and scenario analyses

Rule applied

Under CNS RECIST (RECIST 1.1), a patient with non-target lesions only can be classified as complete response (CR), non-CR/non-progressive disease, or progressive disease (PD), but not as partial response (PR). Partial responses can therefore occur only in patients with at least one measurable target lesion. Measurable-disease responder counts were derived from the reported best-overall-response categories and the reported location of complete responses; no other assumption was required. Patients not evaluable remained in the denominator as non-responders.

Table S6a. Derivation of measurable-disease intracranial response counts

Step	HERTHENA-Lung01, HER3-DXd [11]	HERTHENA-Lung02, HER3-DXd [21,24]	HERTHENA-Lung02, platinum–pemetrexed [21,24]
Patients with CNS lesions at baseline (CNS BICR)	30 (no prior brain radiotherapy)	105	95
≥1 measurable target lesion / non-target lesions only	7 / 23	24 / 81	28 / 67
Best overall intracranial response, all patients	CR 9, PR 1, SD (incl. non-CR/non-PD) 13, PD 4, other/NE 3*	CR 13, PR 7, SD 52, PD 27, NE 6	CR 4, PR 7, SD 47, PD 26, NE 11
CRs in non-target-only patients (reported)	8 of 9	13 of 13	4 of 4
CRs in patients with target lesions	1	0	0
PRs (necessarily in patients with target lesions)	1	7	7
Measurable-disease responders / denominator	2/7 (29%)	7/24 (29%)	7/28 (25%)

*Derived as 30 − (10 + 13 + 4). HERTHENA-Lung01 responses were assessed on CT alone (5 CR, 1 PR), MRI alone (2 CR), or CT with MRI confirmation (2 CR) [11]. CR, complete response; NE, not evaluable; PD, progressive disease; PR, partial response; SD, stable disease.

Table S6b. Scenario analyses for pooled intracranial objective response rate (GLMM)

Scenario	HERTHENA-Lung01; HERTHENA-Lung02	Pooled icORR (95% CI)
Base case (derived counts)	2/7; 7/24	32% (25–41%)
Lower	1/7; 5/24	30% (23–38%)
Higher	3/7; 9/24	35% (27–43%)
Extreme lower	0/7; 4/24	29% (22–37%)
Extreme higher	4/7; 12/24	37% (30–46%)
Both cohorts excluded	—	33% (25–43%)

Table S6c. HERTHENA-Lung02 risk ratio for intracranial response in measurable disease (HER3-DXd vs platinum–pemetrexed), varying each arm by ±2 responders

HER3-DXd \ control	5/28	7/28 (base)	9/28
5/24	1.17 (0.38–3.55)	0.83 (0.30–2.29)	0.65 (0.25–1.67)
7/24 (base)	1.63 (0.59–4.48)	1.17 (0.48–2.85)	0.91 (0.40–2.07)

9/24	2.10 (0.81–5.42)	1.50 (0.66–3.42)	1.17 (0.55–2.46)

Wald 95% confidence intervals on the log scale. All scenarios include 1. Code and data: https://doi.org/10.17605/OSF.IO/3RFQ7.
